# ScRNA-seq revealed an immunosuppression state and tumor microenvironment heterogeneity related to lymph node metastasis in prostate cancer

**DOI:** 10.1186/s40164-023-00407-0

**Published:** 2023-05-23

**Authors:** Shiyong Xin, Xiang Liu, Ziyao Li, Xianchao Sun, Rong Wang, Zhenhua Zhang, Xinwei Feng, Liang Jin, Weiyi Li, Chaozhi Tang, Wangli Mei, Qiong Cao, Haojie Wang, Jianguo Zhang, Lijin Feng, Lin Ye

**Affiliations:** 1 Department of Urology, Shanghai East Hospital, School of Medicine, Tongji University, No.150, Ji-mo Rd, Pu-dong new District, Shanghai, 200120 China; 2 Department of Urology, The First Affiliated Hospital and College of Clinical Medicine of Henan University of Science and Technology, Luoyang, 471003 China; 3 Department of Urology, Putuo People’s Hospital, School of Medicine, Shanghai, China; 4 School of Pharmacy, Inner Mongolia Medical University, Hohhot, 010000 China; 5 School of Pharmaceutical Sciences, Sun Yat-sen University, Guangzhou, 510006 China; 6 School of Pharmaceutical Sciences (Shenzhen), Shenzhen Campus of Sun Yat-sen University, Shenzhen, 518107 China; 7 Department of Pathology, The Third Affiliated Hospital of Henan University of Science and Technology, Henan, 471003 China; 8 Department of Central Laboratory, Zhengzhou University, Luoyang Central Hospital, Luoyang, 471003 China; 9 Department of Pathology, Jing’an District Zhabei Central Hospital, No.619, Zhonghuaxin Road, Shanghai, 200070 China

**Keywords:** Prostate Cancer, Metastasis, Single-cell RNA sequencing, Tumor Microenvironment, Immunosuppression

## Abstract

**Background:**

Metastasis is a crucial aspect of disease progression leading to death in patients with prostate cancer (PCa). However, its mechanism remains unclear. We aimed to explore the mechanism of lymph node metastasis (LNM) by analyzing the heterogeneity of tumor microenvironment (TME) in PCa using scRNA-seq.

**Methods:**

A total of 32,766 cells were obtained from four PCa tissue samples for scRNA-seq, annotated, and grouped. InferCNV, GSVA, DEG functional enrichment analysis, trajectory analysis, intercellular network evaluation, and transcription factor analysis were carried out for each cell subgroup. Furthermore, validation experiments targeting luminal cell subgroups and CXCR4 + fibroblast subgroup were performed.

**Results:**

The results showed that only EEF2 + and FOLH1 + luminal subgroups were present in LNM, and they appeared at the initial stage of luminal cell differentiation, which were comfirmed by verification experiments. The MYC pathway was enriched in the EEF2 + and FOLH1 + luminal subgroups, and MYC was associated with PCa LNM. Moreover, MYC did not only promote the progression of PCa, but also led to immunosuppression in TME by regulating PDL1 and CD47. The proportion of CD8 + T cells in TME and among NK cells and monocytes was lower in LNM than in the primary lesion, while the opposite was true for Th and Treg cells. Furthermore, these immune cells in TME underwent transcriptional reprogramming, including CD8 + T subgroups of CCR7 + and IL7R+, as well as M2-like monocyte subgroups expressing tumor-associated signature genes, like *CCR7, SGKI, and RPL31*. Furthermore, STEAP4+, ADGRF5 + and CXCR4+, and SRGNC + fibroblast subgroups were closely related to tumor progression, tumor metabolism, and immunosuppression, indicating their contributions in PCa metastasis. Meanwhile, The presence of CXCR4 + Fibroblasts in PCa was confirmed by polychromatic immunofluorescence.

**Conclusions:**

The significant heterogeneity of luminal, immune, and interstitial cells in PCa LNM may not only directly contribute to tumor progression, but also indirectly result in TME immunosuppression, which may be the cause of metastasis in PCa and in which MYC played an role.

**Supplementary Information:**

The online version contains supplementary material available at 10.1186/s40164-023-00407-0.

## Introduction

Prostate cancer (PCa) is a common malignant tumor of the urinary system. Its incidence in the male population is second only to lung cancer, accounting for 10% of all male malignant tumors [1]. PCa can be treated with radical surgery or radiation in the early stages, while advanced PCa is mainly treated with endocrine therapy. Eventually, it will progress to castration-resistant PCa. Metastasis is a key cause of disease progression and death in patients with PCa. Lymph nodes and bones are common metastatic sites for PCa [2]. Unfortunately, the exact mechanism causing PCa metastasis has not been fully elucidated. Tumors are highly complex and their occurrence, development, metastasis, and other processes are inseparable from their continuous interaction with the microenvironment. Microvessels, fibroblasts, mesenchymal cells, immune cells, cytokines, and chemical factors that are secreted by various cells in the tumor microenvironment (TME) together form a complex network that regulates tumor occurrence and progression [3]. However, little is known about the surrounding environment of tumor cells, especially the relationship between TME and metastasis.

Tumor metastasis is a complex process that involves multiple steps, including the shedding of tumor cells from the primary tumor, penetration of the basement membrane, survival in blood vessels or the lymphatic system, and proliferation in distant organs. Tumor-infiltrating immune cells, such as CD8 + T and NK cells, are important components of TME that can kill tumor cells as well as promote tumor development [4]. As one of the vital steps in the process of tumor metastasis, tumor cells have to overcome the direct damage caused by tumor immune and metabolic factors [5]. During tumor invasion and metastasis, the dissociation, invasion, migration, and adhesion of tumor cells are closely correlated with changes in TME [4, 6]. TME and tumor cells interact, TME provides nutrients and a suitable environment for tumor growth, and tumor cells in turn constantly change the surrounding environment, including the characteristics of stromal and immune cells. Furthermore, the inflammatory mediators secreted by the modified stromal cells, cytokines, and extracellular matrix degradation participants can also promote tumor development and metastasis. The microenvironment component serves as fertile soil for tumor cell proliferation and progression and acts as a weapon that blunt the immune system attack, which may be the reason that tumor cells can escape immune surveillance and initiate tumor invasion and metastasis [7, 8]. PCa is a malignant tumor with significant heterogeneity, including inter-tumor, intra-tumor, and clinical heterogeneity, which is manifested by spatial and clonal genomic diversity [9]. At present, genomics and transcriptomics studies have revealed the subtypes of PCa characterized by mutations and abnormal transcription [10–12]. However, undifferentiated data were obtained after averaging a large number of cell populations using traditional large-scale sequencing methods. In contrast, single-cell sequencing has the highest resolution and can analyze transcriptional information at the cellular level, which does not only retain the heterogeneity information within tissue, but also analyzes the transcriptional differences among different cell species. In 2009, researchers studied the transcriptome status of four-cell blastomeres in mice for the first time at the single-cell level. Since then, single-cell sequencing technology has been widely used in tumors [13–15]. At present, elucidating the mechanisms related to tumor metastasis remains challenging, due to the lack of understanding of heterogeneity in TME of metastatic tumors. Furthermore, molecular mechanisms related to PCa metastasis have not been fully revealed, likely because many current studies have ignored the role of metastatic microenvironment in PCa. As a revolutionary tool, single-cell sequencing can not only reveal cellular uniqueness with high precision, but can also provide groundbreaking insights into the heterogeneity, evolution, and metastasis of PCa, as well as elucidate the relationship between the tumor and the immune system.

In our study, scRNA-seq was performed on tissue samples from primary lesions and lymphatic metastases of PCa to investigate the heterogeneity of TME through subpopulation analysis of acquired cells, InferCNV of luminal cells, and gene set variation analysis (GSVA) of cell subsets. Moreover, differentially expressed genes (DEGs) and their functional enrichment between the primary lesions and lymphatic metastases were further analyzed. Cell trajectories and cellchat were traced to investigate cell differentiation and communication among different cell groups in TME. Our study aimed to characterize transcriptome landscape differences between the primary lesions and lymphatic metastases and explore the mechanism related to lymphatic metastasis in PCa.

## Methods

### Tissue samples and cell lines

Two pairs of tissue samples (from primary tumors and iliac vascular lymph node metastases (LNM)) were obtained from two patients with PCa who underwent laparoscopic radical prostatectomy at the Shanghai East Hospital after postoperative confirmation of the diagnosis by a pathologist. PCa tissue microarray chips used in the validation experiments were from the Ye Lin Research Group of East Hospital Affiliated with Tongji University. Paraffin sections from 13 cases of patients with PCa combined with lymphatic metastasis were collected for polychromatic tissue immunofluorescence. All experimental procedures were approved by the Ethics Committee of Shanghai East Hospital.

### scRNA-seq analysis

Raw sequence read quality was assessed using FastQC Software. The clean scRNA-seq reads were mapped to the human reference genome hg38 using Cell Ranger software (v.4.0) for all of the samples.The software was downloaded from 10x Genomics web site. Expression matrixes were loaded into R v.4.0.3 using the function Read10X in Seurat (v.4.1.0) and then merged together by column.Empty wells were distinguished from barcoded cells using UMI count distributions.We used DropletUtils to distinguish cells from empty droplets containing only ambient RNA.

The cyclone function implemented in the Rpackage scran was used to score Cell cycle stag,and the Seurat Rpackage was employed to normalize expression values for total UMI counts per cell. For clustering analysis, We considered mitochondrial features and individual donor effects as a source of unwanted variation and were regressed out using the Seurat package. We fitted the mean variance relationship for each sample to avoid selecting for genes with highly variable between-sample effects. Scree plots and Jackstraw permutation tests were used to determine significant principal components. Cluster-level quality control was performed after the standard Seurat clustering pipeline using the following functions in order: FindNeighbors with the first 20 PCs and FindClusters with resolution 1, otherwise default settings. Clusters with average UMI counts of less than 100 were removed.

### scRNA-seq differential expression analysis and cluster marker detection

The R package MAST was used to perform all single-cell differential gene expression analyses and Likelihood ratio tests was performed to identify DEGs between two conditions. Benjamini–Hochberg multiple testing correction was used to estimate p.adjust.We consider Genes with FDR < 5% were significantly differentially expressed. To detect cluster marker genes, cells from each cluster were compared against all other cells in the experiment.The MAST algorithm was used for statistical testing via the Seurat wrapper function FindAllMarkers, with default parameters for filtering out genes below a minimum logfc of 0.25.

### Cell subset annotation by SingleR

The Seurat R package was used to convert scRNA-seq data into Seurat objects. Cell-level quality control analysis was performed to filter cells by (1) total UMI counts of no more than 1,000; (2) gene numbers no higher than 200; or (3) mitochondrial gene percentage of > 20%. The expression level of each gene in each cell was normalized using the NormalizeData function and the LogNormalize method. Scale factor of 10,000 was used to remove the influence of sequencing library size, which converted expression values from UMI counts to ln [10,000 × UMI counts/total UMI counts in cell + 1]. All individual samples were integrated in Seurat using the canonical correlation analysis (CCA) pipeline to remove batch effects. The ‘Select Integration Features’ function was applied to choose the features ranked by the number of datasets. Next, the ‘Find Integration Anchors’ function was utilized to select 2,000 anchors between different samples using the top 50 dimensions from CCA to specify the neighbor search space. ‘IntegrateData’ was then applied to integrate the datasets using the pre-computed anchors and the integrated dataset was scaled using ‘ScaleData’. PCA and uniform manifold approximation and projection (UMAP) dimension reduction based on the top 20 principal components was performed. The identified clusters were visualized on a 2D map produced with the t-distributed t-SNE or UMAP method. Then, the cells were clustered using Seurat’s Find Neighbors with dimensions 1–20 and FindClusters with a resolution of 0.5. The FindAllMarkers functions were used for detection of marker gene expression, followed by the SingleR package and the CellMarker dataset to annotate the cell types. The SubsetData function was also used to extract subclusters for downstream analysis, and UMAP analysis was performed using the RUNUMAP function.

### Cancer cell malignancy recognition by InferCNV

InferCNV refers to the inference of copy number alterations (CNAs) from tumor single-cell RNA sequencing data. InferCNV is used to explore tumor single-cell RNA-Seq data to identify evidence of large-scale chromosomal CNAs in somatic cells, such as expansion or deletion of whole chromosomes or large segments of chromosomes. By comparing this information with a reference set of “normal” cells, the intensity of gene expression at different locations in the tumor genome was explored to pinpoint chromosomal amplification or deletion. Eventually, the relative expression intensity on each chromosome can be determined using a heatmap. It then often becomes apparent which regions of the tumor genome are over- or under-expressed compared to normal cells. Therefore, cancer cell malignancy can be inferred from the degree of chromosomal copy number variation.

### Pathway enrichment analyses

Pathway enrichment analyses were performed using the clusterProfiler Rpackage and we used org.Hs.eg.db Rpackage to map gene identifiers. DEGs were tested individually for overrepresentation by computation of enrichment P values (the enricher Rfunction, default parameters).Benjamini–Hochberg correction was used to adjust Hypergeometric P values in each case for multiple testing.The enrichment results were visualized as dot plots using the enrichPlot function and ggplot2 Rpackage.

To score individual cells for pathway activities, we used the Rpackage AUCell. First, we used AUCell_buildRankings function to compute gene expression rankings in each cell with default parameters.pathway database was downloaded from Misgdbr Rpackage.Then, AUCell_calcAUC function was performed to score each cell,AUC values represent pathway activities for each cell.

### Trajectory analysis

The developmental pseudo-time was determined using the Monocle 2 package. The raw count was first converted from Seurat object into CellDataSet object utilizing the importCDS function in Monocle. The differentialGeneTest function in the Monocle 2 package was used to select ordering genes (qval < 0.01), which were likely to be informative for the ordering of cells along the pseudo-time trajectory. The dimensional reduction clustering analysis was carried out using the reduceDimension function, followed by trajectory inference from the orderCells function using default parameters. Gene expression was plotted with the plot_genes_in_pseudo-time function to track changes over pseudo-time.

### GSVA

To perform GSVA, the GSEABase package (version 1.44.0) was used to load the gene set file downloaded and processed from the KEGG database (https://www.kegg.jp/). GSVA was carried out using standard settings to assign pathway activity estimates to individual cells as implemented in the GSVA package (version 1.30.0). Differences in pathway activities scored per cell were calculated with the LIMMA package (version 3.38.3).

### SCENIC analysis

The SCENIC analysis was performed using the motif database for RcisTarget and GRNboost (SCENIC version 1.1.2.2, which corresponds to RcisTarget 1.2.1 and AUCell 1.4.1) with the default parameters. Briefly, transcription factor (TF) binding motifs over-represented on a gene list were identified using the RcisTarget package. The activity of each regulon group in each cell was scored using the AUCell package. To evaluate cell type specificity of each predicted regulon, the regulon specificity score based on the Jensen-Shannon divergence was calculated to measure the similarity between two probability distributions.

### Cell-cell interactions

“CellChat” in R package was used to perform cell-cell communication analysis (http://www.cellchat.org/). CellChat infers and analyzes intercellular communication networks from scRNA-seq data using network analysis and pattern recognition based on manually curated databases that consider known structural compositions of ligand-receptor interactions. Seurat objects, including count matrix and clustering results for each dataset, were imported to CellChat.

### Immunohistochemistry (IHC)

The expression of MYC, CCL5, EEF1A2 was detected using the SP kit (Zhongshan Jinqiao, Shanghai, China) according to the manufacturer’s instructions.Paraffin sections were dewaxed, hydrated, and rinsed with tap water. Tissue samples were repaired using the corresponding antigen tris-EDTA thermal repair. Peroxidase blocking reagent (H_2_O_2_) was added to the sections, incubated at room temperature for 10 min, and rinsed with phosphate-buffered saline (PBS) three times for 3 min each time. Then, primary antibody of MYC (Thermo Fisher, MA1-980, 1:500), CCL5(Thermo Fisher, 710001,1:400), and EEF1A2(Abcam, ab212172, 1:500) was added to the sections dropwise and incubated overnight at 4 °C. After removing the PBS, secondary antibody was added to the sections, and the samples were incubated at room temperature for 15 min. The samples were then rinsed in PBS three times for 3 min each time. After removing PBS, freshly prepared DAB chromogenic agent was added to the sections for chromogenic development for about 5 min. The samples were then stained with hematoxylin, differentiated with 1% hydrochloric acid ethanol, dehydrated with gradient ethanol, made transparent with xylene, and sealed using neutral gum.

### Immunofluorescence

The concentration of Lncap was diluted to 5 × 10^3^/mL and inoculated into small confocal dishes. The cells were removed from the 37 °C incubator when the cell density reached 50%. The samples were washed with PBS three times for 10 min each time. They were then fixed with 4% formaldehyde (Biyuntian, P0099) at room temperature for 1 h, washed with PBS three times, permeabilized with 2% Triton X-100 (Biyuntian, P0096) for 5–10 min, washed with PBS three times, and blocked with 5% BSA at room temperature for 1 h. Primary antibodies for MYC (Thermo Fisher, MA1-980, 1:100), PD-L1 (FineTest, FNab06280, 1:50), CD47 (Bioss, bs-21460R, 1:100), CCL5 (Thermo Fisher, 710001,1:250), and EEF1A2 (Abcam, ab212172, 1:100) were diluted with 1% BSA, added to the samples, and incubated overnight at 4 °C in a humidified chamber (and reheated for 30 min the next day). The fluorescent secondary antibody Goat Anti-Mouse IgG H&L(Alexa Fluor 555)( Abcam, ab150114, 1:200) and Goat Anti-Rabbit IgG H&L(Alexa Fluor 488)( Abcam, ab150077, 1:200) was diluted with 1% BSA to the appropriate concentration, added to the samples, and incubated for 1 h at room temperature in the dark, followed by washing with PBS three times. Finally, cells (Soleipol, C0065) were stained with DAPI for 15 min, washed with PBS, and imaged.

The as-prepared tumor sections were stained according to the instructions of multiplex fluorescence immunohistochemical staining kit (Absin, Catalog No.abs50013) and blocked with TBST containing 5% goat serum before incubation with antibodies. The antibodies involved in experiment include a-SMA (Cell signaling, Catalog No.19,245, diluted at 1:1000), CXCR4-Antibody(4G10)(Santa, Catalog No.sc-53,534, diluted at 1:2000). The nuclei were stained with DAPI before sealing, and all sections were scanned by a fluorescent scanning camera (KFBIO, KF-TB-400).

### Real-time quantitative polymerase chain reaction (qRT-PCR)

The cDNA samples were obtained after total RNA samples were extracted from the cells using TRIzol reagent (Solibo, R1100). Next, qRT-PCR was performed using StepOne (Applied Biosystems, Foster City, CA, USA). GAPDH was selected as the endogenous control for mRNA. The primer sequences used in the experiments are shown in Table 1.

**Table 1:** Primer sequences used in qRT-PCR

Gene	Forward primer (5’ to 3’)	Reverse primer (5’ to 3’)
MYC	AATGAAAAGGCCCCCAAGGTAGTTATCC	GTCGTTTCCGCAACAAGTCCTCTTC
CD47	TGCGGATCAGCTCAGCTACTA	GTTTTGTGCCTCCATATTAG
PD-L1	TCACGTCTCCAAATGTGTATCACTTTG	ATGAGGATATTTGCTGTCTTTATATTCATG
GAPDH	ACGGATTTGGTCGTATTGGG	CCTGGAAGATGGTGATGGGATT

### Western blot

Proteins were extracted from LNCap cells after treatment with different drugs. Total protein samples were separated by sodium dodecyl sulfate-polyacrylamide gel electrophoresis (10% gel), transferred to polyvinylidene fluoride membranes (PVDF, Millipore, Bedford, MA, USA), blocked with 5% skim milk powder (Shengon Biotechnology, Shanghai, China) for 1 h at room temperature, washed three times with TBST, and then incubated with primary antibodies for MYC (Thermo Fisher, MA1-980, 1:500), PD-L1 (FineTest, FNab06280, 1:1000), and CD47 (Bioss, bs-21460R, 1:500) overnight at 4 °C. On the second day, the PVDF membranes were rewarmed for 30–60 min, washed three times with TBST, and incubated with secondary antibody (1:500, Shenggong Biotechnology, Shanghai, China) for 1 h at room temperature. Chemiluminescence reagent was then used to observe the protein bands (Shanghai TianNeng Technology Co. LTD., Tanon500, China).

### Transwell assay

First, 3 × 10^5^ LNCaP cells were distributed into a six-well plate, and shRNA transfection was performed when the cell density reached 70–80%. The next day, 12 mg/mL of matrigel were spread on the upper surface of a Transwell chamber (Corning, New York, 354,234, USA), diluted 1:4 with serum-free medium, and placed in an incubator at 37 °C for 2 h to solidify. The cells were digested and collected after 24 h of transfection and then resuspended at a concentration of 1 × 10^6^ cells/mL in serum-free medium. Next, 100–200 µL of cells were seeded into the upper Transwell chamber and 500 µL of complete medium containing 10% fetal bovine serum were added into the lower chamber. The 24-well plates were then incubated in a 5% CO_2_ incubator at 37 °C. After 48 h, the cells were fixed with 4% paraformaldehyde for 1 h, washed with PBS three times, stained with 0.1% crystal violet (Solibol G1064) for 30 min at room temperature, and washed with PBS three times. Upper chamber cells were then wiped off, imaged, and photographed under an inverted microscope (Eclipse Ti, Nikon, Japan).

### EDU

The target cell concentration was adjusted to 3 × 10^5^/ well and inoculated into 6-well plate.Transfection was performed when the cell density reached 70 ~ 80%0.48 h after transfection, the transfected cells were replaned and cultured overnight.On the second day, equal volume was added with preheated 2x EDU working solution (20µM), and the cells were incubated for 2 h.Then, it was performed and analyzed according to EDU cell proliferation assay instructions (Biyuntian, Shanghai, China).

### Statistical analysis

The statistical analysis was performed using SPSS 21.0 software (IBM, Armonk, NY, USA). Continuous data were expressed as mean ± standard deviation (SD). The significance of differences was determined using the unpaired or paired Student’s t-test as indicated, and differences with P < 0.05 were considered statistically significant.

## Results

### TME cell composition in primary lesions and LNM

**Fig. 1:**
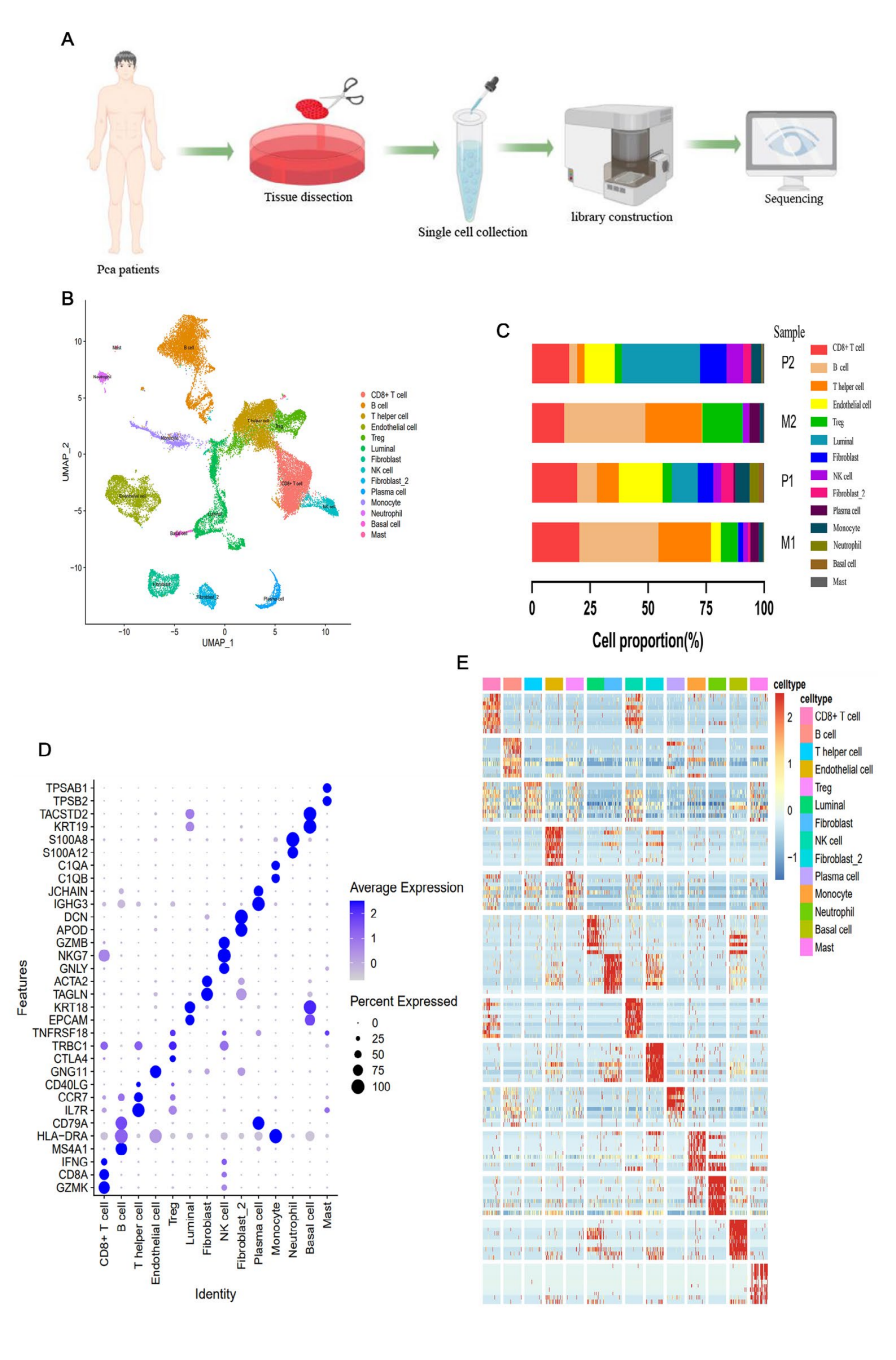
Fourteen cell types in PCa were identified by scRNA-seq. (**A**). Study flow chart; (**B**). Main cell clusters in PCa tissue demonstrated using uniform manifold approximation and projection (UMAP) analysis are colored and labeled according to their featured gene expression profiles. (**C**). Cell numbers and percentages of each cluster in each sample; (**D**). Marker gene expression for each cell type, where dot size and color represent percentage of marker gene expression (pct. exp) and averaged scaled expression (avg. exp. scale) value, respectively; (**E**). Heatmap generated based on expression levels of top ten marker genes in each cluster

The scRNA-seq analysis was performed on four tumor samples from two patients with PCa to determine their cellular composition (Fig. 1A). Single-cell transcriptomes from a total of 32,766 cells were obtained, of which 16,717 cells were from the primary lesion and 16,049 cells were from LNM (Tables 2 and 3). UMAP analysis identified 14 major groups based on genetic profiles and typical marker genes of the cells (Fig. 1B and D; Supplementary Material 10–11). Their specific contents were as follows: (1) CD8 + T cells with high expression of GZMK, CD8A, and IFNG; (2) luminal cells specifically expressing KRT18 and EPCAM; (3) fibroblasts with high expression of ACTA2 and TAGLN; (4) NK cells with high expression of GZMB, NKG7, and GNLY; and (5) monocytes specifically expressing HLA-DRA, C1QA, and C1QB, as well as marker genes in other cell types (Fig. 1D). The results showed the expression profiles of the top 10 highly expressed genes in each cell type, as well as the cell proportion of each cell type (Fig. 1C and E, Supplementary Material 12). Notably, all cell types were present in each primary lesion sample, while basal cells and neutrophils were nearly absent in metastatic lesions (Fig. 1C, Tables 2 and 3). Significant differences in TME cell composition implied obvious heterogeneity between primary lesions and lymphatic metastases. Basal cell subpopulation in our study was not found in lymphatic metastasis lesions, which is consistent with previous findings on basal cell loss and luminal cell expansion in PCa [16].

**Table 2:** The number of cells in each cell population

CD8 + T cell	B cell	T helper cell	Endothelial cell	Treg	Luminal	Fibroblast	NK cell	Fibroblast_2	Plasma cell	Monocyte	Neutrophil	Basal cell	Mast
5843	6685	5001	3233	2543	3091	1576	1133	880	748	1203	471	230	129

**Table 3:** The cell number of each cell population in each sample

	CD8 + T cell	B cell	T helper cell	Endothelial cell	Treg	Luminal	Fibroblast	NK cell	Fibroblast_2	Plasma cell	Monocyte	Neutrophil	Basal cell	Mast
P2_M	1718	2881	1908	358	611	18	190	172	86	301	161	23	0	12
P2_P	2217	961	1077	2158	456	1268	767	380	606	99	699	440	210	56
P9_M	1056	2660	1849	22	1312	14	7	201	7	342	112	0	0	28
P9_P	852	183	167	695	164	1791	612	380	181	6	231	8	20	33

### Transcriptional heterogeneity and InferCNV of luminal cells

**Fig. 2:**
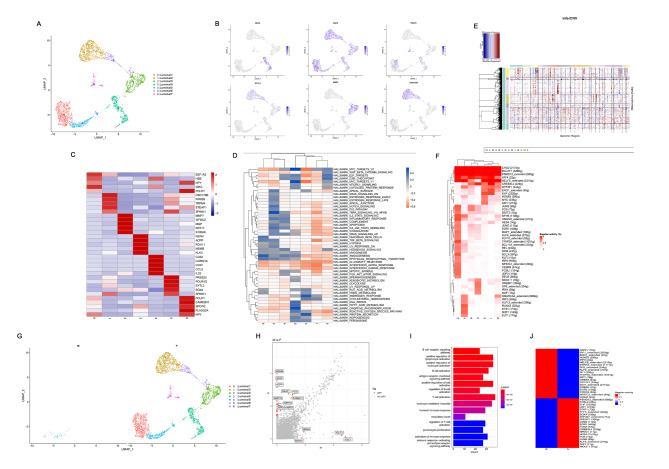
Heterogeneity and CNV analysis of luminal cells; (**A**). Seven main luminal subgroups identified using UMAP analysis; (**B**). Feature plots for marker genes; Color legend shows log1p normalized gene expression levels; (**C**). Heatmap of average expression for top five DEGs among seven subgroups. Color legend indicates normalized gene expression levels among subgroups; (**D**). GSEA heatmap for 50 hallmark gene sets in MSigDB database among seven luminal subclusters; (**E**). Hierarchical heatmap showing large-scale CNVs in seven luminal subgroups; (**F**). Differential analysis heatmap of transcriptional regulators among seven luminal subgroups; (**G**). Luminal subgroups in primary and metastatic lesions identified using UMAP analysis; (**H**). Scatter plot of DEGs between primary lesion and lymphatic metastases. Top 10 DEGs are labeled in red; (**I**). Functional enrichment DEG analysis in primary lesion and lymphatic metastases. (**J**). Heatmap of activated transcription factors in lymphatic metastases and primary lesions. Red indicates high activity, and blue shows low activity

According to previous studies, luminal and basal cells are the possible initiating cells of PCa [17]. UMAP analysis of luminal cells identified a total of seven subgroups (Fig. 2A) and demonstrated gene expression patterns in different luminal cell subgroups (Fig. 2B, Supplementary Material 13). The top five merker genes in each subgroup of luminal cells were also demonstrated (Fig. 2C), which were *EEF1A2, HBB, IGKC, NPY, and FOLH1* in the luminal 1. HBB and IGKC were highly expressed in the luminal 5, while NPY and FOLH1 were highly expressed in the luminal 7 (Fig. 2C). GSVA showed that MYC and oxidative phosphorylation signaling pathways were enriched in the luminal 1 subgroup, while TNF-α signaling pathway activity was enhanced in the luminal 3 subgroup. In addition, the luminal 4 subgroup had high protein secretion and androgen response pathway enrichment score, while the E2F and G2M signaling pathways were enriched in the luminal 6 subgroup. The luminal 7 subgroup demonstrated enhanced activity in the angiogenesis signaling pathway (Fig. 2D).

**Fig. 3:**
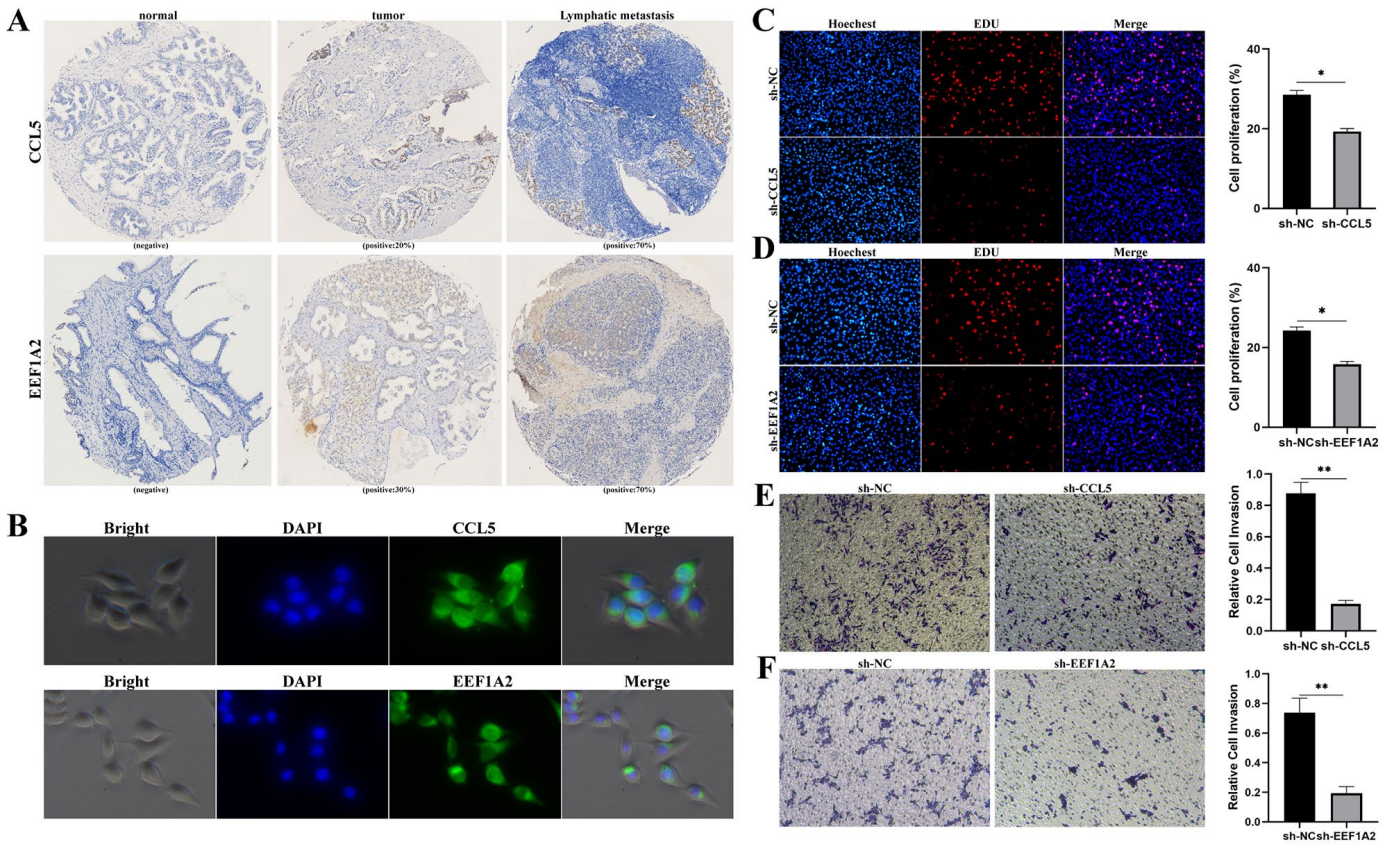
EEF2 + and FOLH1 + luminal cells existed in PCa. (**A**). Immunohistochemistry analysis of CCL5 and EEF1A2 through PCa tissue microarray; (**B**). Immunofluorescence of CCL5 and EEF1A2 in LNCap. (**C-D**). EDU showed the cell proliferation capacity of LNCaP after CCL5 and EEF1A2 down-regulation. (**E–F**). Metastatic ability of LNCAP cells was analyzed using transwell assay after down-regulation of CCL5 and EEF1A2

The InferCNV algorithm was used to identify chromosomal CNAs to further verify the presence of malignant cells in the seven luminal subgroups. As described previously [18], this method can identify typical PCa genomic alterations [10, 19], including the chromosome 8q gain and chromosome 8p, 13, and 16q loss. Chen et al. found that only an inflection point was observed in a few PCa samples, which can separate putative malignant cells from non-malignant cells in PCa. However, this distinction was not so precise in other samples [17], which may be because some localized PCa tumors have been shown to have a silent genome [20]. Furthermore, previous DNA sequencing studies have revealed that 0–50% of PCa genomes have CNAs and that CNAs are also a prognostic factor for PCa [21]. Moreover, the subclone CNA load far exceeds the clonal load in most PCa cases [22]. The InferCNV results of our study are shown in Fig. 2E. All cells in the luminal subgroups 1 and 5, as well as 3 and 7, were clustered together. All cells in the other subgroups were grouped separately (Fig. 2E). In the CNA heatmap, red and blue represent excessive and low gene expression values in the fragment chromosomes, respectively. This means that the darker the red color, the higher the degree of chromosome amplification, and the darker the blue color is, the higher the degree of chromosome loss. Therefore, the study results showed that luminal subgroups 1 and 5, as well as 3 and 7, had a much higher degree of chromosome CNAs than other clusters. Therefore, cells in the luminal subgroups 1 and 5, as well as 3 and 7, may be malignant ones (Fig. 2E). Furthermore, luminal subgroups 1 and 5 only appeared in metastatic lesions, implying that these two subgroups may contain cells with metastatic ability among the malignant cells (Fig. 2G). The DEGs and their functional enrichment between primary lesion and LNM were further analyzed to reveal specific gene expression patterns of LNM in PCa (Fig. 2H-I, Supplementary Material 14). The results showed that the main enriched functional DEG pathways were immune-related, such as regulation of leukocyte and lymphocyte activity and B cell activity (Fig. 2I), indicating that tumor immunity may contribute to lymphatic metastasis in PCa. Furthermore, the transcriptional regulators of luminal cells were analyzed and compared between primary lesions and LNM (Fig. 2F and J). JDP2, IRF1, ENO1, HOXB2, IRF8, NELFE, ESRRA, SP8, KLF9, SF1, TFAP2A, IRF7, CREB3, GTF2F1, MXI1, CREB5, EGR3, FOXO3, NFATC3, and CREM were all activated in LNM, but not in the primary lesions (Fig. 2F and J; Supplementary Fig. 1A–G). In particular, the expression of NELFE in lymphatic metastases was significantly higher than that in the primary lesion. It has been reported that NELFE can promote the invasion and metastasis in many cancers. Therefore, the above results demonstrated that these TFs may be involved in lymphatic metastasis of PCa (Fig. 2J, Supplementary Fig. 1H). Subsequently, markers gene EEF1A2 and CCL5 with specificity in EEF2 + and FOLH1 + luminal subgroups were selected for immunohistochemical staining on our PCa tissue chips, and the results showed negative results in normal tissues.The positive expression of some PCa cells in the primary and lymphatic metastases suggests the existence of these two subgroups of cells in PCa (Fig. 3A). Immunofluorescence demonstrated that MEEF1A2 and CCL5 were all expressed in LNCap cells (Fig. 3B). Down-regulating the expression of MEEF1A2 and CCL5 in LNCap cells could significantly reduced the ability of cell proliferation and metastasis ((Fig. 3C-F).

### MYC may promote lymphatic metastasis by promoting tumor cell progression and immunosuppression

**Fig. 4:**
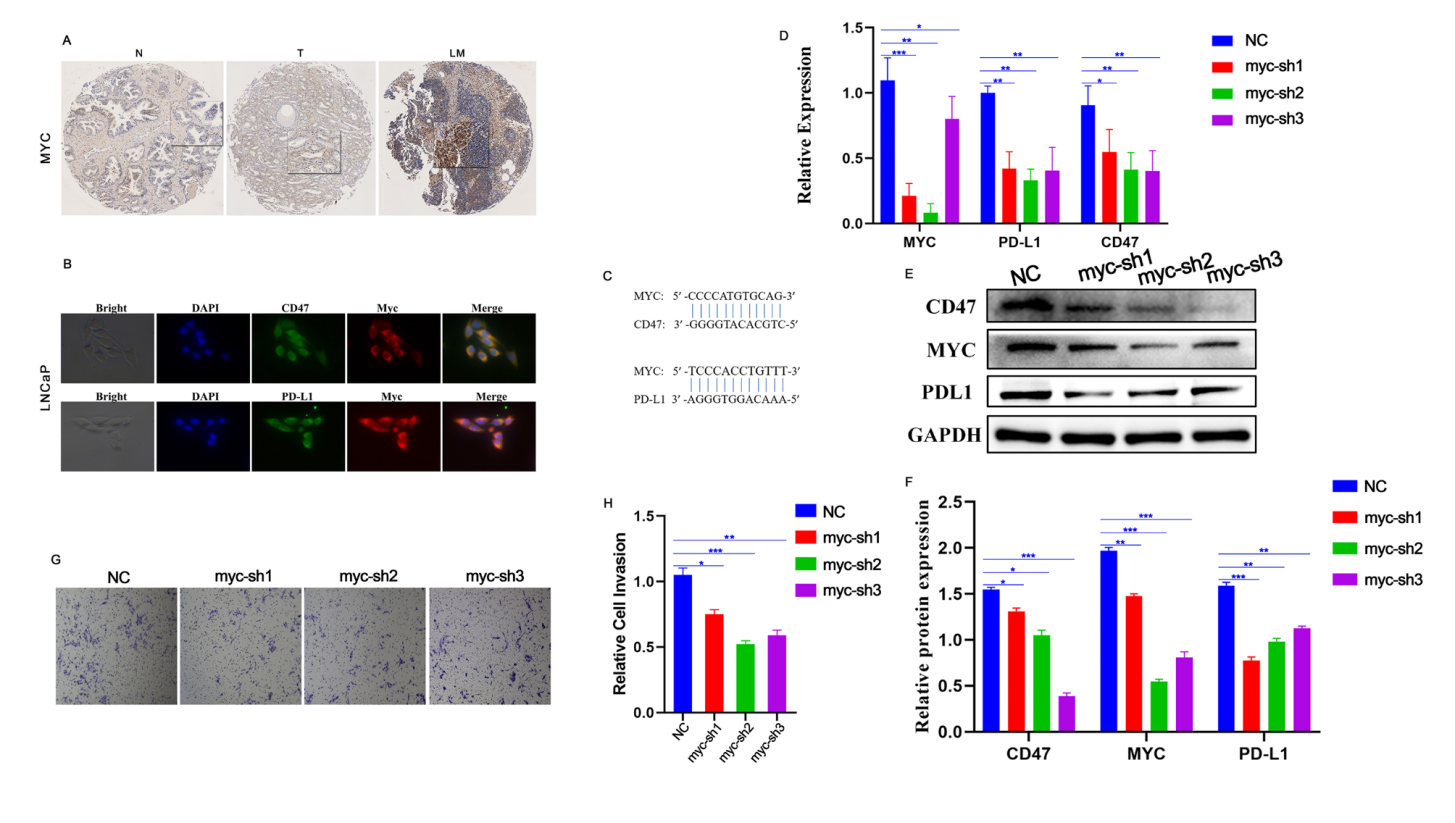
MYC drives tumor progression through CD47 and PD-L1. (**A**). Immunohistochemistry analysis of MYC through PCa tissue microarray; N: Normal prostate tissue, T: Prostate cancer tissue, LM: lymphatic metastases; (**B**). Immunofluorescence of MYC, PDL1, and CD47 in LNCap; (**C**). Bioinformatics analysis of binding sites of MYC to PDL1 and CD47; (**D**). MYCmRNA, PDL1mRNA, and CD47mRNA expression in LNCaP after MYC down-regulation. (**E–F**). MYC, PDL1, and CD47 western blot protein expression analysis in LNCaP; (**G–H**). Metastatic ability of LNCAP cells analyzed using transwell assay after down-regulation of MYC.

The luminal subgroups 1 and 5 appeared in the lymphatic metastases. The MYC pathway was also shown to be enriched in these cells and was therefore investigated further. MYC is a well-known oncogene that belongs to a class of nuclear gene family that includes C-MYC, N-MYC, and L-MYC [23]. A study from Science found that MYC inhibition in mouse tumors reduced the CD47 and PD-L1 mRNA and protein levels and enhanced antitumor immune responses [24]. Therefore, MYC can promote tumor progression directly as well as give rise to tumor immunosuppression, which can indirectly result in tumor progression by regulating CD47 and PD-L1. In our study, the microarray IHC results showed that MYC was expressed in the nucleus and cytoplasm, and its expression in PCa tissues was significantly stronger than that in normal prostate tissues. Importantly, the MYC expression in LNM was significantly higher than that in the primary lesions (Fig. 4A), indicating a relationship between MYC and PCa metastasis. The immunofluorescence results demonstrated that MYC, PDL1, and CD47 were all expressed in LNCap cells, while PDL1 and CD47 were mainly expressed in the cytosol (Fig. 4B). Results obtained from the NCBI (https://www.ncbi.nlm.nih.gov/) and JASPAR (https://jaspar.genereg.net/) databases showed that the promoter regions of PDL1 and CD47 had binding sites for MYC, demonstrating that PDL1 and CD47 were the target genes of MYC (Fig. 4C). After further down-regulating the expression of MYC in LNCap cells via plasmid transfection, it was found that the mRNA and protein levels of PDL1 and CD47 were significantly down-regulated(P < 0.05) (Figure.4D-F). Moreover, the Transwell results indicated that down-regulation of MYC expression significantly reduced the metastatic ability of LNCap cells (P < 0.05) (Fig. 4G-H).

### Luminal cell trajectory analysis

**Fig. 5:**
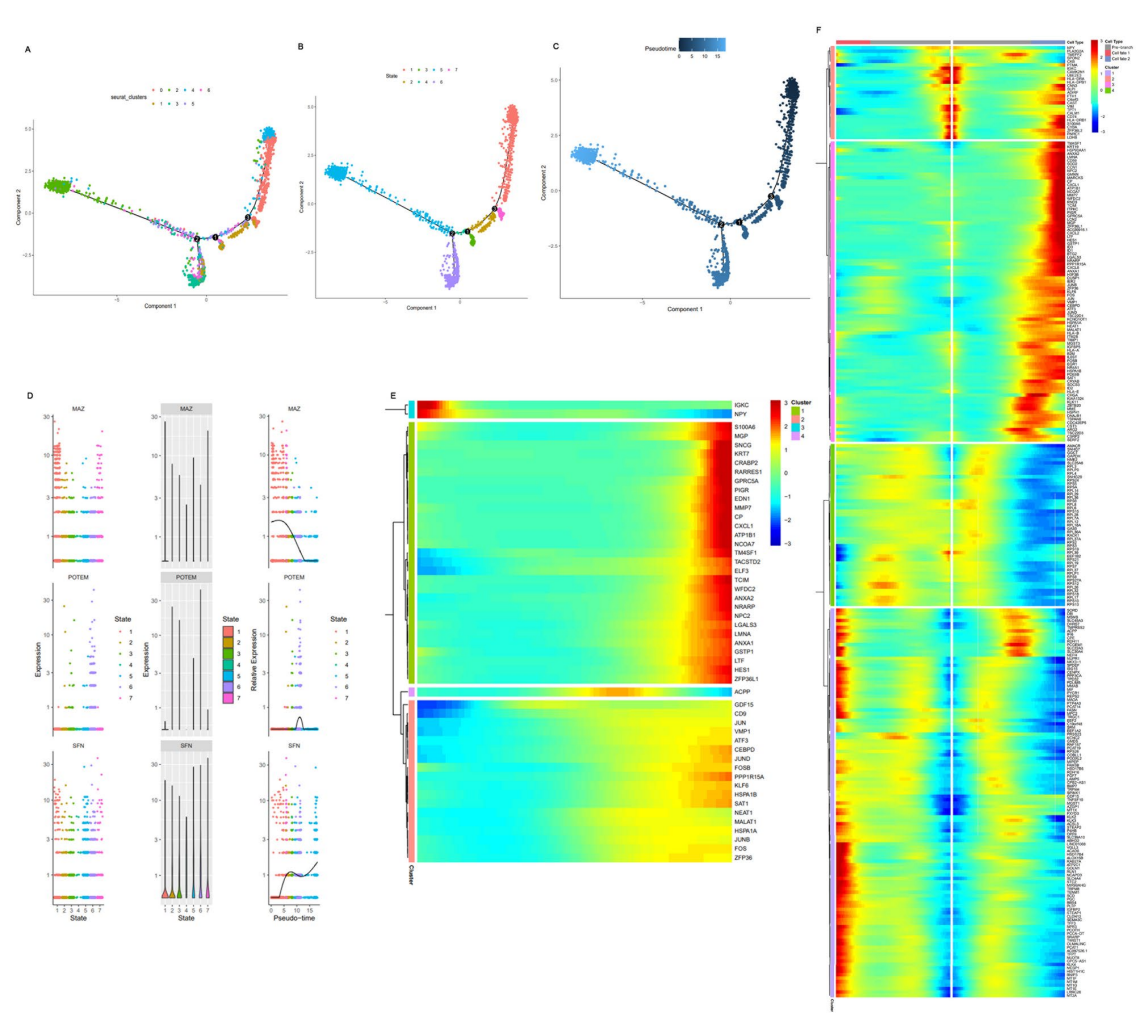
Luminal cell trajectory analysis. (**A–C**). Monocle 2 trajectory plot showing luminal subcluster and state dynamics; (**D**). Three representative genes with different expression patterns in the process of luminal cell differentiation: MAZ, POTEN, and SPN; (**E**). Hierarchical clustering heatmap showing four subclusters of differentially expressed genes along with luminal cell pseudotime. (**F**). Hierarchical clustering heatmap showing four subclusters of differentially expressed genes along with pseudotime for three cell types (from cell fate1 to cell fate2)

The cell trajectory results are shown in Fig. 4, where Monocle 2 divided all luminal cells into seven states (Fig. 5B). Figure 4A shows the differentiation trajectory of cells according to their cluster after dimensionality reduction clustering by Seurat. Clusters 0 and 4 were distributed on the right side of the figure, while clusters 2 and 6 were distributed on the left side of the figure. The other three clusters (1, 3, and 5) were located in the lower part in the middle of the figure (Fig. 5A). Furthermore, the differentiation trajectory of the luminal cells took the second branch points as an important turning point from the right to the left and lower part of the figure (Fig. 5C). Therefore, the overall differentiation of luminal cells occurred from clusters 0, 1, and 4 to clusters 2, 3, and 6. The InferCNV results showed that clusters 0, 2, 4, and 6 were potential malignant cells, while clusters 1, 3, and 5 were non-malignant cells. The two clusters 0 and 4 were metastatic cells, implying that cancer cells with metastatic ability were early occurrence (Fig. 5C, Supplementary Fig. 2). Moreover, the differentiation direction of malignant cells was different from that of the non-malignant cells.

Next, the transcriptome changes during luminal cell differentiation were explored. Representative genes with different expression characteristics were selected to observe their dynamic changes during cell differentiation (Fig. 5C). MYC-associated zinc-finger protein (MAZ) plays a transcriptional regulatory role in some important genes, including MYC, RAS, and CT-1. Moreover, MAZ contributes greatly to the occurrence and development of PCa [25]. MAZ expression first increased and then decreased sharply with cell differentiation time (Fig. 5D). Sialophorin (SPN), also known as LSN and CD43, is a transmembrane salivary glycoprotein. In addition to being present in mature red blood cells and immune cells, SPN has also been found to be expressed in hematopoietic cells [26–28]. Accompanied by luminal cell differentiation, SPN gene expression initially remains unchanged for a short time at baseline, followed by a sharp increase, a mild decline, and a final rapid increase (Fig. 5D). The expression changes of MYC, PDL1, and CD47 in the trajectory process of luminal cell were further investigated. The results showed that MYC expression showed an initial short-term increase, followed by a decrease, return to the baseline level, and a final increase. CD47 showed a sharp increase that was then maintained. Interestingly, the expression of PDL1 (CD274) did not change in the whole cell trajectory process (Supplementary Fig. 3). The above results indicated that MYC and CD47 were involved in the differentiation of luminal cells. Finally, the visualization and clustering results for the characteristic genes expression changes during luminal cell differentiation are shown in Fig. 5E. Subsequently, luminal cells were divided into cell fate 1, pre-branch, and cell fate 2 subsets according to the differentiation process. During the process of luminal cell differentiation from cell fate 1 to cell fate 2, all of the changed genes could be clustered into four categories based on their expression levels (Fig. 5F).

### Heterogeneous characterization of CD8 + T cells in primary and lymphatic metastatic lesions

**Fig. 6:**
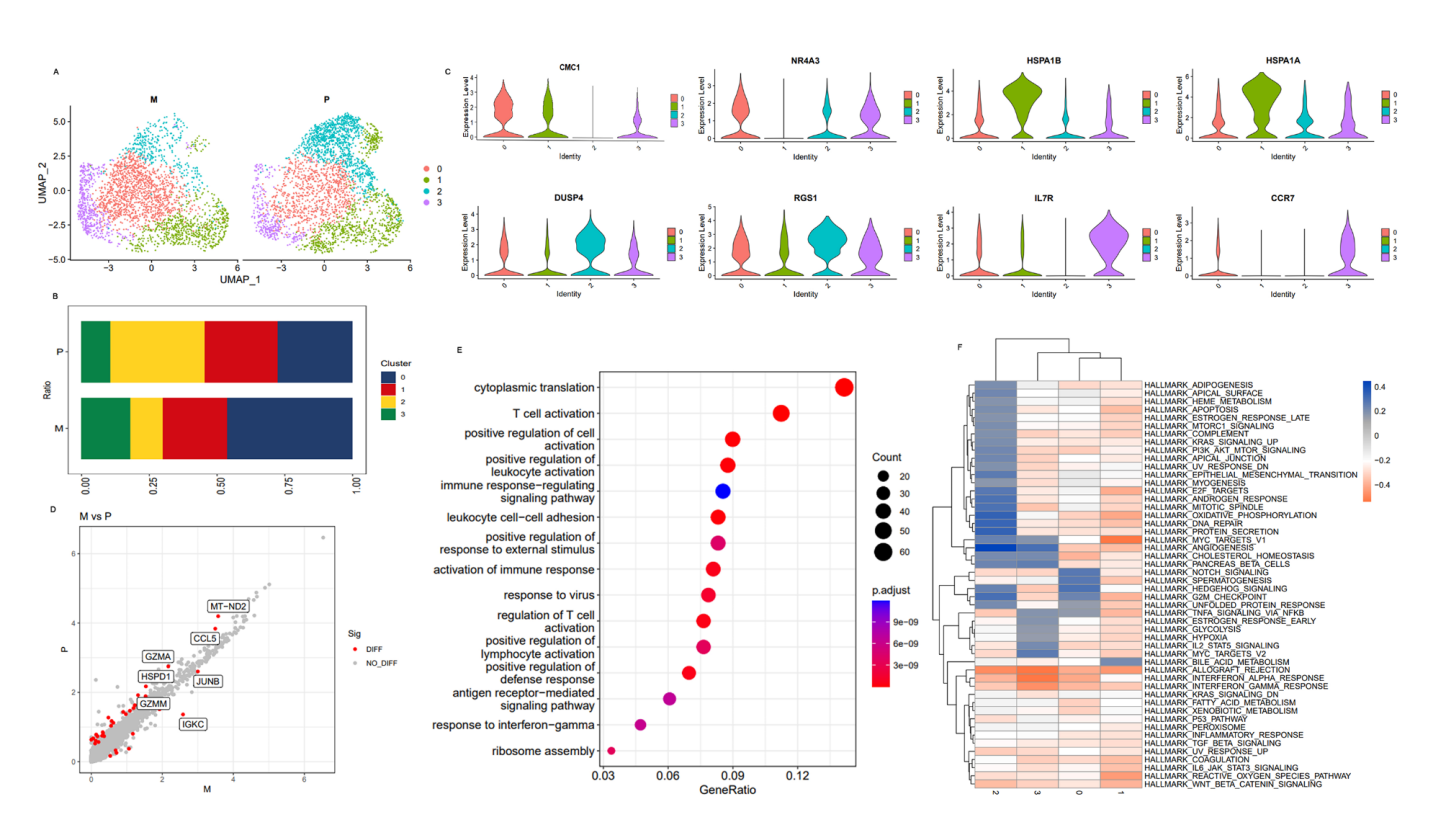
Heterogeneity analysis of CD^+^8 T cells between primary and metastatic lesions; (**A**). Subcluster of CD^+^8 T distribution between primary and metastatic lesions using UMAP-2 analysis; (**B**). Percentage of four CD^+^8 T subclusters between primary and metastasis lesions; (**C)**. Violin plots showing normalized marker gene expression levels across four subclusters of CD^+^8 T cells; (**D**). DEGs in metastasis identified using edgeR package with comparison to primary lesion. Scatter plots showing respective DEG profiles in PCa. Red spots indicate up-regulated genes; green spots indicate no significant gene change; (**E**). Functional DEG enrichment analysis; (**F**). GSEA heatmap of 50 hallmark gene sets in MSigDB database among four CD^+^8 T cell subclusters

High heterogeneity in T cell type composition, gene expression patterns, and functional properties can significantly affect the outcome of T cell-based immunotherapy [29]. In tumor immunity, CD8 + T cells usually have a crucial antitumor role. However, tumor-mediated depletion of T cells prevents CD8 + T cells from exerting normal cytotoxicity, resulting in immunosuppression [30]. In our study, 5,843 CD8 + T cells were identified (3,069 in the primary lesion and 2,774 in the metastatic lesion). The proportion of CD8 + T cells in the metastatic lesion was significantly smaller than that in the primary lesion (Tables 2 and 3), implying the possibility of immunosuppression or more obvious T cell depletion in the metastatic lesion. A total of four subsets of CD8 + T cells were identified using UMAP analysis of CD8 + T cells (Fig. 6A, Supplementary Material 15). Furthermore, the results showed that the proportion of subsets 0 and 3 in the metastatic lesions was significantly higher than that in the primary lesions (Fig. 6C). Moreover, among the marker genes, CCR7 was only expressed in subsets 0 and 3 (Fig. 6C). Studies have found that CCR7 promotes tumor development by promoting tumor cell proliferation and metastasis, encouraging proteolytic enzyme secretion, and inducing angiogenesis and immunosuppression [31–36], which is consistent with our findings, where CD8 + T and other tumor immune cell levels decreased and underwent transcriptional recombination, resulting in significant immunosuppression in lymphatic metastases. In addition, the GSVA results in our study showed that pathways enriched in subsets 0 and 3 included Hedgehog signaling, notch signaling, angiogenesis, and MYC pathways, which are associated with tumor proliferation and progression (Fig. 6F). These results indicated that marker genes expressed by subsets 0 and 3 had the characteristics of tumor genes, which can promote tumor proliferation and metastasis as well as cause tumor immunosuppression. The proportion of subset 2 was also significantly smaller in the metastatic lesion than in the primary lesion (Fig. 6B), and NR4A3, DUSP4, and RGS1 were the marker genes with a higher expression (Fig. 6C). The GSVA results in our study showed that pathways enriched in subset 2 included apoptosis, EMT, oxidative phosphorylation, and DNA repair (Fig. 6F). These results confirmed that marker genes expressed in the subset 2, such as NR4A3, can restrain PCa metastasis by mediating the MT-2 signaling pathway, showing the opposite function to that of CD8 + T cell subsets 0 and 3. These results demonstrated that T cell heterogeneity and immunosuppression in TME may be important causes of LNM in PCa. Further analysis of DEGs between primary lesions and lymphatic metastases showed that IGKC and JUNB were highly expressed in the metastatic lesions. JUNB (JunB proto-oncogene) is a protein-coding gene that can act on the DAP12 receptor in NK cells to regulate the immune response. Primary cutaneous T-cell lymphoma and anaplastic large cell lymphoma are closely related to JUNB [37]. Functional enrichment pathway analysis of DEGs showed that these genes were mainly concentrated in cytoplasmic translation, T cell activation, positive regulation of leukocyte activation, immune response regulation, leukocyte adhesion, and other immune-related pathways (Fig. 6D and E). These results further demonstrated that CD8 + T cells in lymphatic metastasis of PCa had the characteristics of promoting tumor progression and leading to immunosuppression. Principal component analysis of different T cell groups in the immune microenvironment of breast cancer by Azizi et al. revealed that T cells were in a continuous activation and differentiation trajectory, and their phenotypic diversity was jointly determined by various environmental stimuli and T cell receptors [38]. Zheng et al. used the Monocle 2 algorithm to analyze the developmental trajectory of T cells and found that CD8 + T cells had a state transition process from activation to depletion, and that GZMK + subsets were the intermediate states in this transition process [39]. Our trajectory analysis of CD8 + T cells found that most of the CD8 + T cells in metastasis were located in the middle and posterior segment of the whole cell differentiation, and CD8 + T cells showed a high expression of cytotoxic markers GZMM and GZMB (Supplementary Fig. 4, Supplementary Material 15), implying that GZMK + subsets in PCa were intermediate states in the CD8 + T cell transition process.

### Heterogeneity of monocytes in primary and lymphatic metastatic samples

**Fig. 7:**
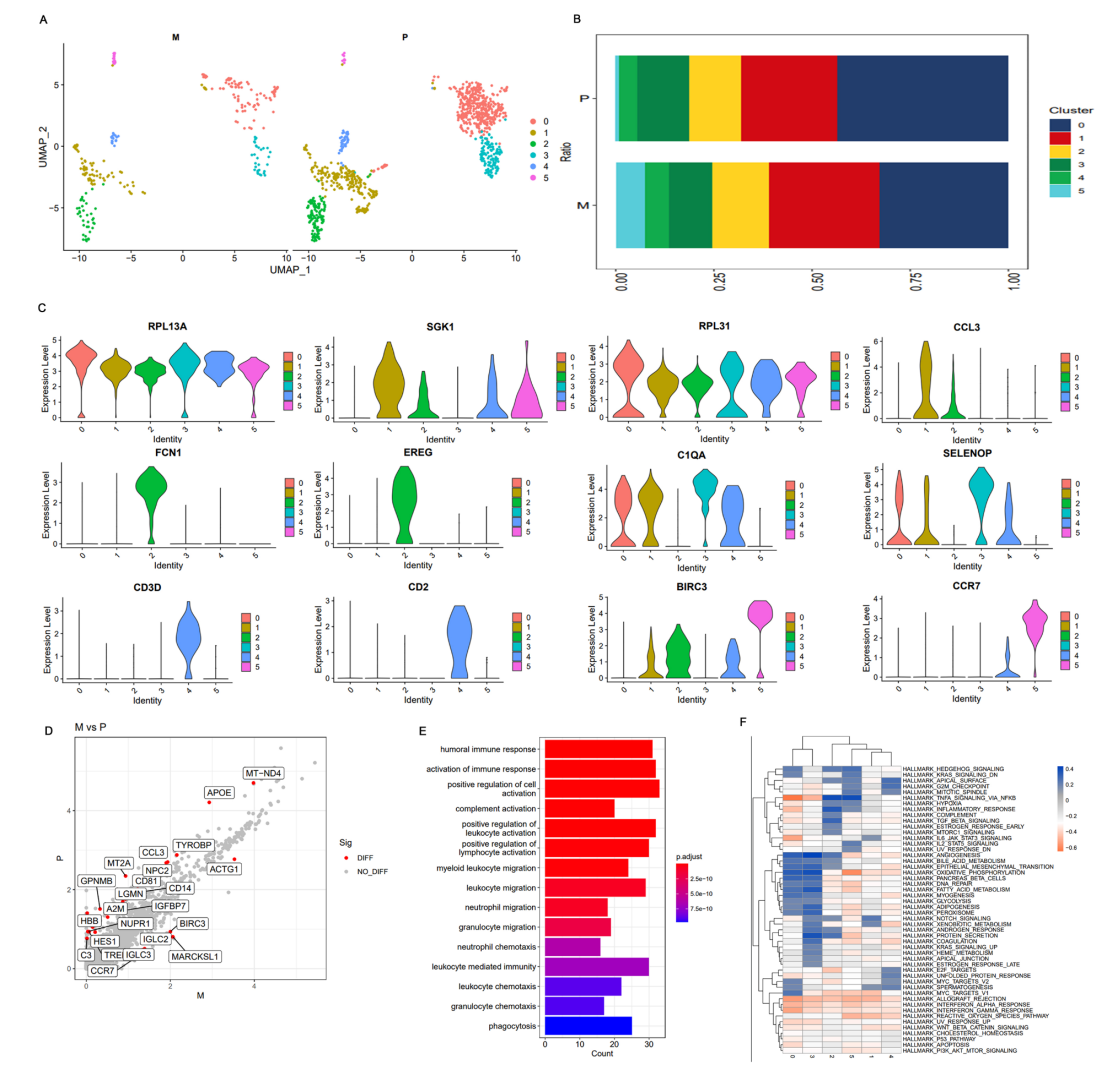
Heterogeneity analysis of myeloid cells in primary and lymphatic metastatic TME of PCa. (**A**). Monocyte distribution subcluster between metastatic and primary lesions using UMAP-2 analysis; (**B**). Mean percentage of monocyte subclusters in primary and metastatic lesions; (**C**). Violin plots showing normalized expression levels of marker genes across monocyte subclusters; (**D**). Metastasis DEGs identified using edgeR package with comparison to primary lesion. Scatter plots showing respective DEG profiles in PCa. Red spots indicate up-regulated genes; green spots indicate no significant change in genes; (**E**). Functional enrichment analysis for monocyte DEGs; (**F**). GSEA heatmap of 50 hallmark gene sets in MSigDB database among monocyte subclusters

Myeloid cells are another important component in TME in addition to lymphoid cells. Nirschl et al. determined the transcriptome of monocytes in the microenvironment of lymph node metastatic melanoma and found that these cells up-regulated the expression of genes related to immune homeostasis, indicating that tumors might use the mechanism of the body regulating autoimmune homeostasis for immune escape [40]. In our study, a total of 1,203 monocyte cells were identified (930 in primary lesions and 273 in metastatic lesions), and the number and proportion of monocyte cells in lymphatic metastatic lesions were significantly smaller than those in the primary lesions (Tables 2 and 3). UMAP analysis of monocyte cells identified a total of six subsets (Fig. 7A, Supplementary Material 16). In addition, cell proportions in subsets 1, 2, 4, and 5 in the metastatic lesions were higher than those in the primary lesions. BIRC3 overexpression was found in all four subsets, while subsets 1 and 2 simultaneously overexpressed the M1 characteristic gene CCL3 (Fig. 7B). Furthermore, GSVA analysis showed that TNF-α signaling, inflammatory response, and TGF-β signaling pathway were enriched in the subsets 1 and 2. In addition to BIRC3, the characteristic genes that promote tumor proliferation and progression, including RPL31 and EREG, were all expressed in subsets 1 and 2 (Fig. 7C, F). The above results demonstrated that subsets 1 and 2 might be the transition stage from M1 to M2, which was consistent with the results reported by Azizi et al. who found that some TAM cell groups in breast cancer highly expressed both the M1 (such as CCL3) and M2 (such as MARCO and NRP2) characteristic genes. This implied that TAM differentiation in TME was also a continuous and progressive process, rather than two discrete states as traditionally believed [38]. Moreover, GSVA showed that E2F, MYC, G2M, TNF-α-signaling, and Hedgehog signaling pathways were enriched in subsets 4 and 5. The above results suggested that subsets 4 and 5 had the characteristics of M2. However, the proportion of cells in subsets 0 and 3 in the primary lesion was significantly higher than that in lymphatic metastases, suggesting their possible tumor suppressor function. The characteristic genes SELENOP and C1QA of these two subgroups are closely related to immunity and energy metabolism [41, 42]. In our study, the GSVA results indicated that protein secretion, oxidative phosphorylation, bile acid metabolism, and other metabolism-related pathways were mainly enriched in subsets 0 and 3 (Fig. 7C, F), indicating that these cells may interfere with TME by regulating immunity and metabolism. In addition, monocyte DEGs between primary lesions and lymphatic metastases were further investigated. BIRC3, CCR7, and especially ACTG1 in the lymphatic metastases promote the proliferation and metastasis of PCa [43]. Functional enrichment pathway analysis of DEGs showed that they were mainly concentrated in immune response activation, leukocyte migration, positive regulation of cell activation, and other immune-related pathways (Fig. 7D, E; Supplementary Material 16). Cell trajectory analysis showed that most of the monocyte cells in the metastatic lesions were located in the middle of the whole cell differentiation and differentiated toward the first and second branches below the main line (Supplementary Fig. 5).

Furthermore, the study results demonstrated that the number and proportion of NK cells and neutrophils, like CD8 + T cells, were much smaller in LNM than in primary PCa lesions. Functional enrichment analysis of DEGs showed that they were mainly enriched in immune, metabolic, and tumor proliferation-related pathways (Supplementary Fig. 6, Supplementary Material 17–18). Treg cells can accumulate in tertiary lymphoid organs and can inhibit antitumor immunity. Our results showed that the number and proportion of Treg and Th cells in lymphatic metastases of PCa were significantly higher than those in the primary lesions (Supplementary Fig. 7, Supplementary Material 19–20). Cell clusters expressing immunoglobulin and B cell-specific TFs were the most numerous in breast cancer microenvironment. B cells were divided into two categories: one with center cell/glial cell-expressing characteristics and the other with naive B lymphocyte expression characteristics [44], which shows B cell heterogeneity in the tumor tissue. Similar to the outcomes reported in the literature, our results showed that the number and proportion of B cells in LNM of PCa were significantly higher than those in primary lesions (Supplementary Fig. 8, Supplementary Material 21), which implies that B cells also contribute to PCa metastasis.

### Heterogeneity analysis of fibroblasts

**Fig. 8:**
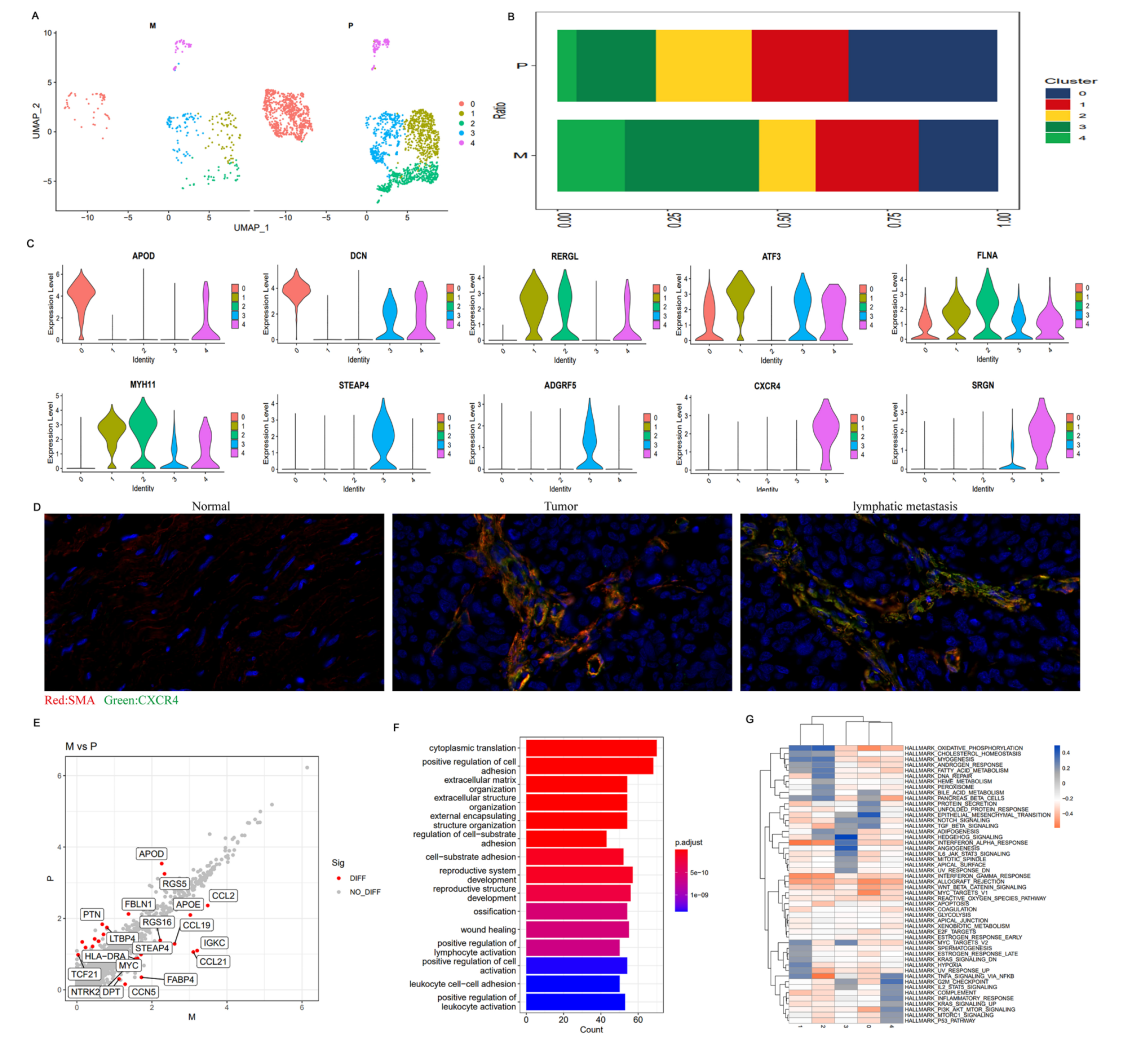
Heterogeneity analysis of fibroblasts in primary and lymphatic metastatic PCa TME. (**A**). Subcluster of fibroblast distribution between metastatic and primary lesions using UMAP-2 analysis; (**B**). Mean percentage of fibroblast subclusters in primary and metastatic lesions; (**C**). Violin plots showing normalized expression levels of marker genes across fibroblast subclusters; (**D**). Tissue immunofluorescence showing CXCR4 + Fibroblasts in normal tissue, primary lesions and lymphatic metastases; red fluorescence representing SMA and green fluorescence representing CXCR4.(**E**).DEGs in metastasis were identified using edgeR package with comparison to primary lesion. Scatter plots showing respective DEG profiles in PCa. Red spots indicate up-regulated genes; green spots indicate no significant change in genes; (**F**). Functional enrichment analysis of fibroblast DEGs; (**G**). GSEA heatmap of 50 hallmark gene sets in MSigDB database among fibroblast subclusters

In addition to immune cells, the TME also contains cancer-associated fibroblasts (CAFs), vascular endothelial cells, extracellular matrix, and other non-immune cell components, which also affect the functional status of tumor immune microenvironment. Tirosh et al. combined scRNA-seq data with data from the TGCA database and found a series of CAF-expressed genes strongly correlated with T cell infiltration, including chemokine ligand 2 (C-X-C motif chemokine ligand 2 (CXCL2), C-C motif chemokine ligand 19 (CCL19), and other chemokines. Immune regulatory genes, such as PD-L2, and complement factor 3 were strongly correlated with the infiltration of CD8 + T cells. CAFs may be involved in the regulation of T cell tumor infiltration [18]. A total of 2,456 fibroblast cells (2,066 in primary lesions and 390 in metastatic lesions) were identified in our study (Tables 2 and 3). UMAP analysis of fibroblasts revealed a total of five subsets (Fig. 8A, Supplementary Material 22). The percentages of cells in subsets 3 and 4 were significantly higher in lymphatic metastatic lesions than in primary lesions, indicating that subset 3 and 4 cells were associated with tumor progression and metastasis (Fig. 8B). Further study of the characteristic genes expressed in these two subsets revealed genes co-expressed in other subgroups, such as DCN, ATF3, and FLNA (Fig. 8C). More importantly, STEAP4 and ADGRF5 specifically expressed in subgroup 3 and 4 showed a significant overexpression of CXCR4 and SRGN (Fig. 8C), which were closely related to tumor metastasis or immunosuppression in TME. Our multicolor immunofluorescence staining on paraffin sections of 13 cases of PCa combined with lymphatic metastasis indicated that CXCR4 was positive in the primary tumor tissue and lymphatic metastases, and the positive rate was higher in the metastatic lesions than in the primary lesions, which was consistent with the results of single cell sequencing. It suggested that there were CXCR4 + bibroblasts in PCa TME (Figure. 8D). By binding CXCL12, CXCR4 plays an important role in mediating immune and inflammatory responses, regulating hematopoiesis, inducing angiogenesis, tumor invasion, metastasis, and other physiological and pathological processes [45, 46]. The GSVA results of our study showed that Hedgehog signaling, angiogenesis, and G2M were significantly enriched pathways in fibroblasts of subsets 3 and 4 (Fig. 8G), and that activation of these pathways can cause tumor progression. The above results demonstrated that fibroblast subsets 3 and 4 with high expression of STEAP4, ADGRF5, CXCR4, and SRGNC underwent significant functional alterations caused by transcriptional reprogramming, which was closely related to tumor progression and metastasis, tumor metabolism, and immunosuppression, implying that these fibroblasts had the ability to promote PCa metastasis and progression. Further analysis of the DEGs and their functional enrichment between primary lesions and lymphatic metastases of PCa (Fig. 8E–F), especially the DEGs highly expressed in lymphatic metastases, showed that the DEGs highly expressed in metastases included immune-related genes (CCL21, CCL19, CCL2, and MYC), genes associated with metabolism (STEAP4, FABP4, DPT, and APOE), and genes associated with tumor proliferation and progression (RGS, CCN, and MYC). Functional enrichment analysis of DEGs showed that these DEGs were mainly enriched in cytoplasmic translation, positive regulation of cell adhesion, extracellular matrix organization, and positive regulation of lymphocyte activation involved in the regulation of extracellular matrix and immune response. In conclusion, these results demonstrated that there was remarkable heterogeneity among fibroblasts in the TME of PCa. Some fibroblast subgroups expressed tumor and immune-related genes, which could affect tumor progression and remold tumor cells and immune cells in TME, thus promoting the metastasis of PCa.

### Cell-to-cell interaction network analysis revealed cell-to-cell communication in primary and lymphatic metastatic TME of PCa

**Fig. 9:**
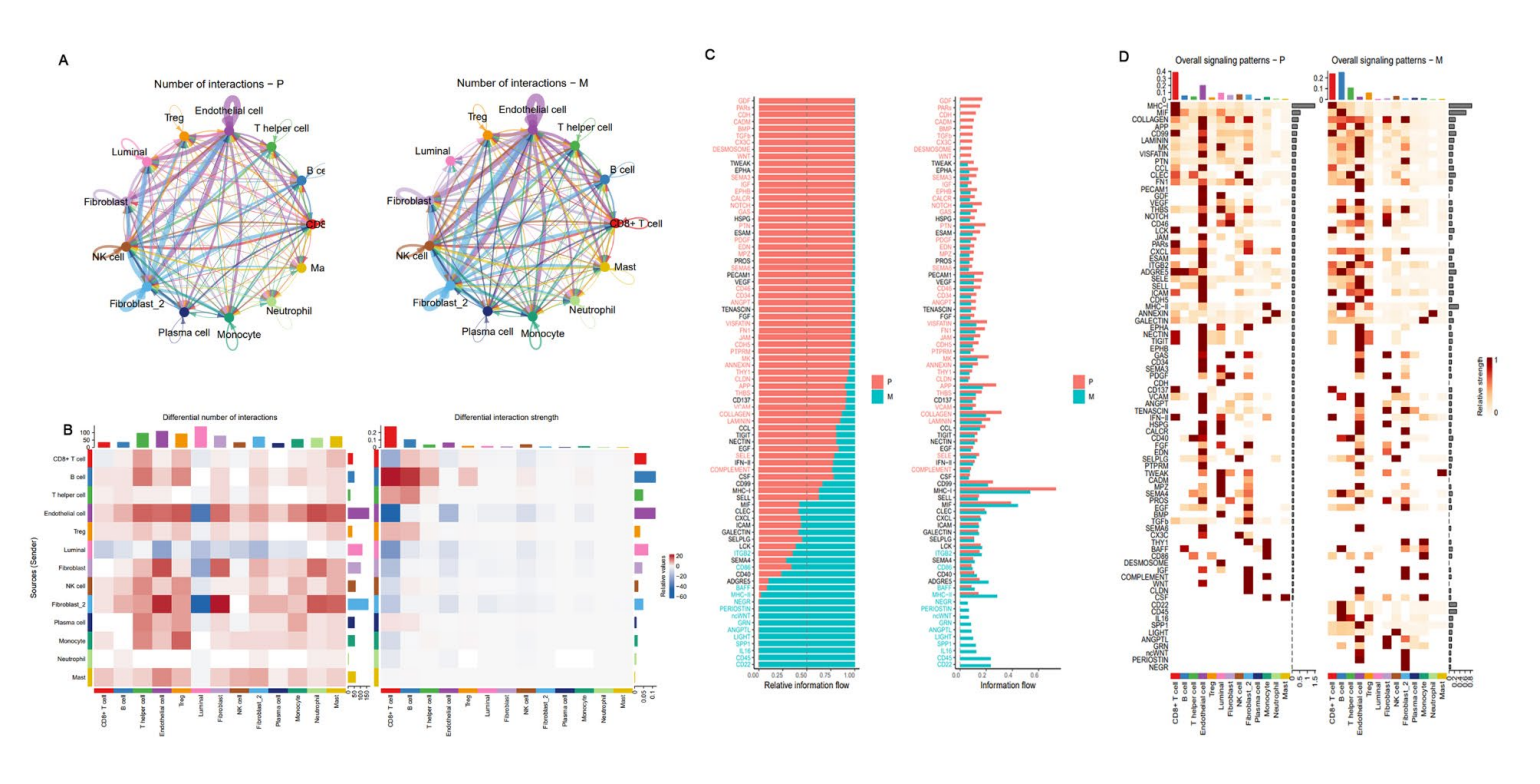
Cell-to-cell communication in primary and lymphatic metastatic PCa TME. (**A**). Cell communication network in primary and lymphatic metastatic lesions; (**B**). Heatmap showing cell interaction pathways in primary and lymphatic metastatic lesions identified for each cell type. (**C**). Bar plots of signaling axes ranking using overall information flow differences in interaction networks between primary and lymphatic metastatic lesions. Top signaling pathways with red-colored labels are more enriched in primary lesion, middle pathways with black-colored labels are equally enriched in lymphatic metastatic and primary lesions, and bottom pathways with green-colored labels are more enriched in lymphatic metastatic lesions. (**D**). Overall signaling patterns for each cell type in primary and lymphatic metastatic lesions

To determine the differences in intercellular communication between primary and metastatic TME in PCa, CellChat analysis was performed to model cell-cell interactions among luminal/immune/stromal cells in the TME of PCa. Based on the gene expression of receptor-ligand pairs, the cell interaction pathway and intensity in the primary lesions and LNM samples were inferred to obtain the cell interaction network. As a result, the number of inferred interactions in the lymphatic metastases was greater than that in the primary lesions, while the interaction strength was the opposite. Furthermore, the interaction between mesenchymal and immune cells was tighter in lymphatic metastases, while luminal cell communication was weakened compared to that in the primary lesions (Fig. 9A, B, Supplementary Fig. 9A–D). Next, 82 activated signaling pathways were analyzed and identified in PCa, 63 of which were shared by the primary and lymphatic metastatic lesions (Fig. 9C, D), and nine were unique to the primary lesions, including GDF, PARs, CDH, CADH, BMP, TGFb, CX3C, DESMOSOME, and WNT, among which GDF and PARs were more active. Ten signaling pathways only existed in the lymphatic metastases, including CD22, CD45, IL16, SPP1, LIGHT, ANGPTL, GRN, ncWNT, PERIOSTIN, and NEGR, in addition to the classical ncWNT pathways that can promote tumor progression. CD45 and CD22 were the most active (Fig. 9C, D). In addition, the output signals of CD + 8 T cells, endothelial cells, luminal cells, NK cells, and fibroblasts were more active in the primary lesion, while the output signals of CD + 8 T cells, endothelial cells, Th cells, and Treg cells were more active in the metastatic lesions, implying the possible related mechanism of immunosuppressive state in PCa metastasis (Supplementary Fig. 9E). Together, these results indicated that intercellular communication in the lymphatic metastatic lesion might lead to an immunosuppression state in TME and tumor progression.

## Discussion

PCa is a highly heterogeneous malignancy composed of multiple cell types and is characterized by spatial and clonal genomic diversity. Under the context of traditional sequencing technologies, significant differences in gene expression among various cell populations in tumor tissue will be masked. However, scRNA-seq detects gene expression at the individual cell level, which can better identify differential genes, allowing for the identification of unexpected biological features of different cell types []. At present, scRNA-seq has been used to reveal the heterogeneity of various tumor types and PCa scRNA-seq has been reported in the literature. For example, studies have revealed the tumor characteristics of PCa using scRNA-seq of liquid biopsies and cultured PCa cells [48,49]. As the origin of PCa, luminal cells may play a decisive role in the progression and metastasis of PCa. Chen et al. analyzed the transcriptomic data of 36,424 cells from 13 PCa samples and identified potentially malignant epithelial cells. Moreover, they found that multiple transcriptome programs related to tumor progression were activated and verified that cancer cells had the ability to change the transcriptome of T cells [17].We identified seven different types of luminal cell subgroups. The luminal subgroup 1 and 5, as well as 3 and 7, were identified as potentially malignant epithelial cells based on their CNA levels. Furthermore, only luminal subgroup 1 and 5 were found in the lymphatic metastatic lesions. In addition, cell trajectory analysis showed that the occurrence time of luminal cells in LNM was consistent with that of primary tumor cells, implying that malignant cells in LNM were already present in the initial stage of PCa, and the cells with metastatic ability were a specific population in PCa cells. GSVA results showed that MYC was enriched in the luminal subset 1, indicating that these luminal cells may have characteristics of strong tumor growth, metastasis, immune evasion, and metabolic ability. Casey SC et al. found that MYC contributed greatly to antitumor immunity by regulating CD47 and PD-L1 [24].Consistent with previous study, our results demonstrated that MYC, as an oncogene, does not only directly participate in tumor progression and metastasis, but also induces an immunosuppressive microenvironment through PDLI or CD47 to promote metastasis in PCa.

CD8 + T cells play an important antitumor role in tumor immunity, but tumor-mediated depletion of T cells prevents CD8 + T cells from exerting their cytotoxic effects [30]. Myeloid cells were another important component in tumor immune microenvironment besides lymphoid cells. Nirschl et al. determined the transcriptome of monocytes in the microenvironment of lymph node metastatic melanoma and found that such cells up-regulated the expression of genes related to immune homeostasis [50]. Our results showed that the numbers and proportions of immune cells with tumor immune function, such as CD8 + T cells, NK cells, and monocytes, were significantly smaller in lymphatic metastases than in primary lesions of PCa, implying immunosuppression in the TME of PCa. Among the four CD8 + T cell subgroups, CCR7^+^ and IL7R^+^ CD8 + T cells (subsets 0 and 3) were significantly more common in lymphatic metastases than in primary lesions. Studies have also found that silencing CCR7 by siRNA or miRNA led to reduced metastasis and tumor growth in PCa models, indicating that CCR7^+^CD8^+^ T cell subgroups had the ability to promote PCa metastasis, and CD8^+^ T cells underwent transcriptional recombination and showed the characteristics of tumor cells [34]. Further, cytokine IL-7 and its receptor IL-7R were found to be essential for B cell development, differentiation and the survival of naive T cells, as well as for the generation and maintenance of memory T cells [51]. IL-7R can mediate potential tumor-promoting functions in solid cancers [52, 53]. Moreover, pathways enriched in CCR7 + and IL7R + CD8 + T cell subgroups were associated with tumor proliferation and progression, tumor metastasis, and immunosuppression. Therefore, our results demonstrated that CD8 + T cells with CCR7 + and IL7R + had the characteristics of malignant and immune cells, which could lead to tumor progression and immunosuppression. Same as CD8 + T cells in the TME of PCa,our results showed that monocytes underwent significant transcriptional reprogramming, leading to changes in cell function and passivation of their own tumor immunity.

Recent studies have found that CAFs play multiple roles in TME. Our study showed that the proportions of STEAP4 + and ADGRF5 + fibroblasts, as well as CXCR4 + and SRGN + fibroblasts, were significantly higher in lymphatic metastases than that in primary lesions, implying that they were related to PCa progression and metastasis. Studies have shown that STEAP4 can promote PCa cell proliferation [54], and CXCR4 participates in the activation of various pro-cancer regulatory mechanisms, thereby promoting tumor proliferation and metastasis [46]. In addition, SRGN can reprogram aggressive and immunosuppressive TME and regulate the expression of PD-L1 and proinflammatory cytokines in Luad cells [55]. Hedgehog signaling, angiogenesis, and G2M checkpoint pathways were enriched in these fibroblasts, and activation of these pathways could lead to tumor progression. Furthermore, the highly expressed DEGs of fibroblasts in LNM included immune-related genes (CCL21, CCL19, CCL2, and MYC), metabolism-related genes (STEAP4, FABP4, DPT, and APOE), and genes related to tumor proliferation and progression (RGS, CCN, and MYC). In addition, the DEGs were mainly enriched in the pathways involved in the regulation of extracellular matrix and immune response. In conclusion, our results indicated that fibroblasts in the TME of lymphatic metastases were significantly heterogeneous, and some subgroups highly expressed tumor cell- and immune cell-related genes that could modify tumor and immune cells in TME, thus promoting PCa lymphatic metastasis.

In conclusion, our study revealed the transcriptome landscapes and the heterogeneity of luminal cells, tumor infiltrating immune cells, and fibroblasts contributed to the special TME in metastasis of PCa, which was characterized by high cell growth capacity, high levels of immune suppression, and high metabolic status, which was also supported by relevant experiments in our studies. However, our conclusions were based on a limited sample. Therefore, larger-scale scRNA-seq analysis with more PCa samples combined with robust clinical data is needed to further elucidate the exact mechanism of PCa metastasis.

### Electronic supplementary material

Below is the link to the electronic supplementary material.

**Supplementary Fig. 1**. (**A–G**). Transcription factors activated by various luminal cell subsets; (**H**). Transcription factor activity comparison between primary and lymphatic metastatic lesions.

**Supplementary Fig. 2**. (**A**) Luminal cell distribution in primary and lymphatic metastatic lesions demonstrated using uniform manifold approximation and projection (UMAP) analysis. (**B–C**). Monocle 2 trajectory plot showing dynamics of luminal subclusters of primary and lymphatic metastatic lesions; (**D**). Functional enrichment analysis of DEGs in luminal (GO-BP); (**E**). Functional enrichment analysis of DEGs in luminal (GO-CC); (**F**). Functional enrichment analysis of DEGs in luminal (GO-MF); (**G**). Functional enrichment analysis of DEGs in luminal (KEGG).

**Supplementary Fig. 3.** Three representative genes with different expression patterns identified in the process of luminal cell differentiation: (**A**). MYC; (**B**). CD47; and (**C**). PDL1.

**Supplementary Fig. 4**. (**A**). Distribution of CD + 8 T cells in primary and lymphatic metastatic lesions demonstrated using uniform manifold approximation and projection (UMAP) analysis. (**B–C**). Monocle 2 trajectory plot showing CD + 8 T cell dynamics in primary and lymphatic metastatic lesions; (**D**). Functional enrichment analysis of DEGs in CD + 8 T cells (GO-BP); (**E**). Functional enrichment analysis of DEGs in CD + 8 T cells (GO-CC); (**F**). Functional enrichment analysis of DEGs in CD + 8 T cells (GO-MF). (**G**). Functional enrichment analysis of DEGs in CD + 8 T cells (KEGG).

**Supplementary Fig. 5**. (**A**). Monocyte distribution in primary and lymphatic metastatic lesions demonstrated using uniform manifold approximation and projection (UMAP) analysis; (**B–C**). Monocle 2 trajectory plot showing monocyte dynamics in primary and lymphatic metastatic lesions; (**D**). Functional enrichment analysis of DEGs in monocytes (GO-BP); (**E**). Functional enrichment analysis of DEGs in monocytes (GO-CC); (**F**). Functional enrichment analysis of DEGs in monocytes (GO-MF); (**G**). Functional enrichment analysis of DEGs in monocytes (KEGG).

**Supplementary Fig. 6**. (**A–B**). Distribution of neutrophil subclusters between primary and lymphatic metastatic lesions demonstrated using uniform manifold approximation and projection (UMAP) analysis; (**C**). Functional enrichment analysis of DEGs in neutrophils (KEGG); (**D**). GSVA heatmap of 50 hallmark gene sets in MSigDB database among neutrophil subclusters; (**E–F**). Distribution of NK subclusters between primary and lymphatic metastatic lesions demonstrated using uniform manifold approximation and projection (UMAP) analysis; (**G**). Functional enrichment analysis of DEGs in NK cells (KEGG); (**H**). GSVA heatmap of 50 hallmark gene sets in MSigDB database among NK cell subclusters.

**Supplementary Fig. 7**. (**A–B**). Distribution of Treg subclusters between primary and lymphatic metastatic lesions demonstrated using uniform manifold approximation and projection (UMAP) analysis; (**C**). Functional enrichment analysis of DEGs in Treg cells (KEGG); (**D**). GSVA heatmap of 50 hallmark gene sets in MSigDB database among Treg subclusters; (**E–F**). Distribution of Th subclusters between primary and lymphatic metastatic lesions demonstrated using uniform manifold approximation and projection (UMAP) analysis; (**G**). Functional enrichment analysis of DEGs in Th (KEGG); (**H**). GSVA heatmap of 50 hallmark gene sets in MSigDB database among Th subclusters.

**Supplementary Fig. 8**. (**A–B**). Distribution of B cell subclusters between primary and lymphatic metastatic lesions demonstrated using uniform manifold approximation and projection (UMAP) analysis; (**C–D**). Monocle 2 trajectory plot showing B cell dynamics in primary and lymphatic metastatic lesions; (**E**). Functional enrichment analysis of DEGs in B cells (GO-BP); (**F**). Functional enrichment analysis of DEGs in B cells (GO-CC); (**G**). Functional enrichment analysis of DEGs in B cells (GO-MF); (**H**). Functional enrichment analysis of DEGs in B cells (KEGG); (**I**). GSVA heatmap of 50 hallmark gene sets in MSigDB database among B cell subclusters.

**Supplementary Fig. 9**. (**A**). Cell communication network (including number and strength) in PCa; (**B–C**). Plot map showing increased signaling among cell types in primary and lymphatic metastatic lesions; (**D**). Bar chart showing the number of inferred interactions and interaction strength between primary and lymphatic metastatic lesions; (**E**). Outgoing signaling pattern of each cell type in primary and lymphatic metastatic lesions.

Supplementary Material 10: Marker genes of all celltypes.

Supplementary Material 11: Celltypes identified in this study.

Supplementary Material 12: Top 10 DEGs of each celltype.

Supplementary Material 13: DEGs of all luminal subgroups.

Supplementary Material 14: DEGs of luminal cells between primary lesion and LNM.

Supplementary Material 15: DEGs of CD8^+^ T cells.

Supplementary Material 16: DEGs of Monocytes.

Supplementary Material 17: DEGs of NK cells.

Supplementary Material 18: DEGs of Neutrophil cells.

Supplementary Material 19: DEGs of Treg cells.

Supplementary Material 20: DEGs of Th cells.

Supplementary Material 21: DEGs of B cells.

Supplementary Material 22: DEGs of Fibroblasts.

## Data Availability

The data used to support the findings of this study are available from the corresponding authors upon request.

## List of Abbreviations

CCA: Canonical Correlation Analysis
CAFs: Cancer-Associated Fibroblasts
CNAs: Copy Number Alterations
DEGs: Differentially Expressed Genes
GSVA: Gene Set Variation Analysis
IHC: Immunohistochemistry
LNM: Lymph Node Metastasis
MAZ: MYC-Associated Zinc-finger protein
PCa: Prostate Cancer
PVDF: Poly Vinylidene Fluoride membranes
qRT-PCR: Real-Time quantitative Polymerase Chain Reaction
SD: Standard Deviation
SPN: Sialophorin
TF: Transcription Factor
TME: Tumor Microenvironment
UMAP: Uniform Manifold Approximation and Projection
