# Correction: ScRNA-seq revealed an immunosuppression state and tumor microenvironment heterogeneity related to lymph node metastasis in prostate cancer

**DOI:** 10.1186/s40164-024-00517-3

**Published:** 2024-05-20

**Authors:** Shiyong Xin, Xiang Liu, Ziyao Li, Xianchao Sun, Rong Wang, Zhenhua Zhang, Xinwei Feng, Liang Jin, Weiyi Li, Chaozhi Tang, Wangli Mei, Qiong Cao, Haojie Wang, Jianguo Zhang, Lijin Feng, Lin Ye

**Affiliations:** 1 Department of Urology, Shanghai East Hospital, School of Medicine, Tongji University, No.150, Ji-mo Rd, Pu-Dong New District, Shanghai, 200120 China; 2 Department of Urology, The First Affiliated Hospital and College of Clinical Medicine of Henan University of Science and Technology, Luoyang, 471003 China; 3 Department of Urology, Putuo People’s Hospital, School of Medicine, Shanghai, China; 4 School of Pharmacy, Inner Mongolia Medical University, Hohhot, 010000 China; 5 School of Pharmaceutical Sciences, Sun Yat-Sen University, Guangzhou, 510006 China; 6 School of Pharmaceutical Sciences (Shenzhen), Shenzhen Campus of Sun Yat-Sen University, Shenzhen, 518107 China; 7 Department of Pathology, The Third Affiliated Hospital of Henan University of Science and Technology, Henan, 471003 China; 8 Department of Central Laboratory, Zhengzhou University, Luoyang Central Hospital, Luoyang, 471003 China; 9 Department of Pathology, Jing’an District Zhabei Central Hospital, No.619, Zhonghuaxin Road, Shanghai, 200070 China

**Correction: Experimental Hematology & Oncology (2023) 12:49** 10.1186/s40164-023-00407-0

In Fig. 3E, F of this article [1] the authors erroneously used the transwell picture of myc as the result of CCL5 and EEF1A2, resulting in an error in Fig. 3E, F in the published; the figure should have appeared as shown below.

**Fig. 3:**
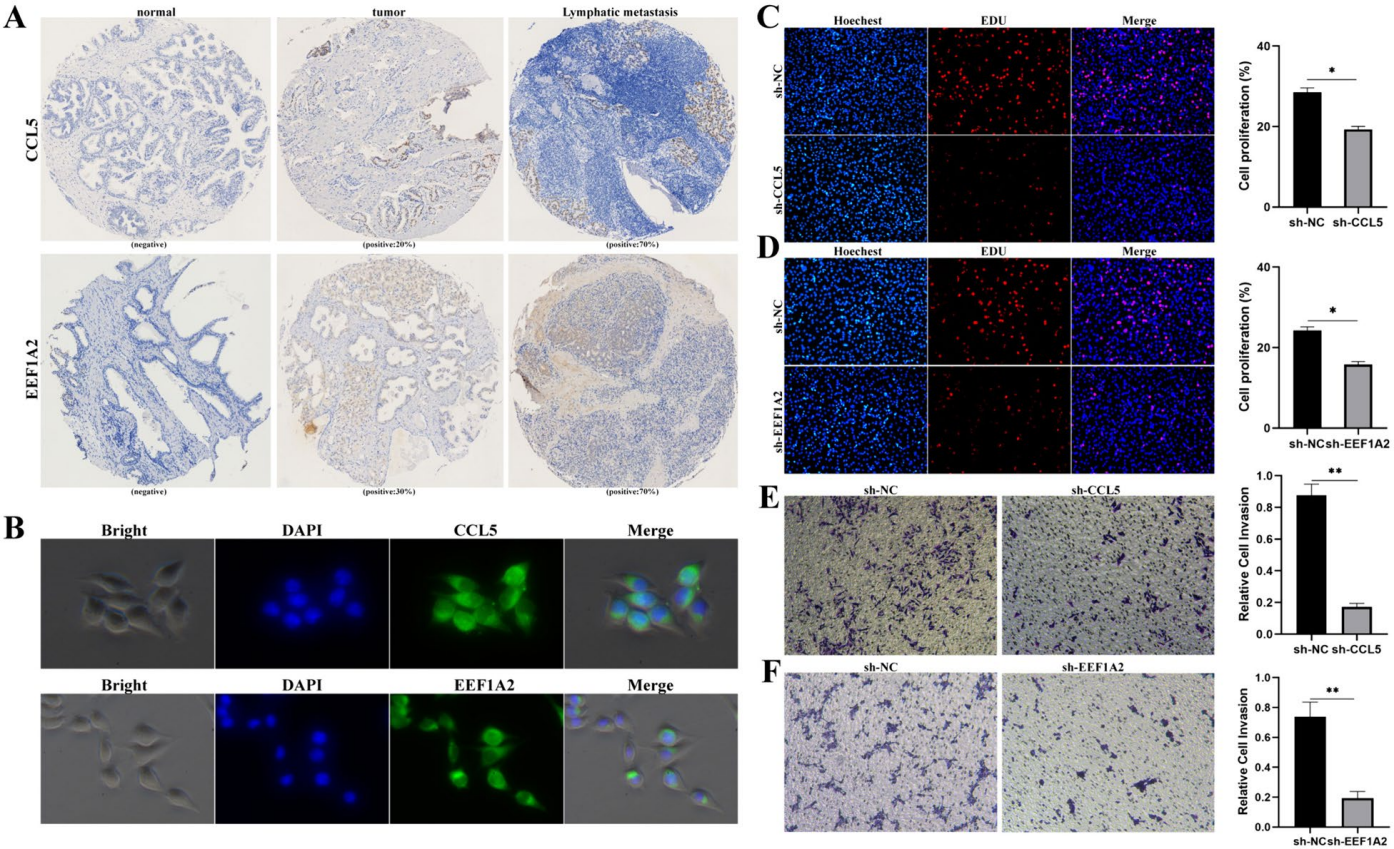
EEF2 + and FOLH1+ luminal cells existed in PCa. **A** Immunohistochemistry analysis of CCL5 and EEF1A2 through PCa tissue microarray; **B** Immunofluorescence of CCL5 and EEF1A2 in LNCap. **C**, **D** EDU showed the cell proliferation capacity of LNCaP after CCL5 and EEF1A2 down-regulation. **E**, **F** Metastatic ability of LNCAP cells was analyzed using transwell assay after down-regulation of CCL5 and EEF1A2

The original article has been corrected.
