# Supplementary figures and images for: ScRNA-seq revealed an immunosuppression state and tumor microenvironment heterogeneity related to lymph node metastasis in prostate cancer
Source: Exp Hematol Oncol. 2023 May 23;12:49. doi: 10.1186/s40164-023-00407-0 (PMC10204220; doi:10.1186/s40164-023-00407-0)

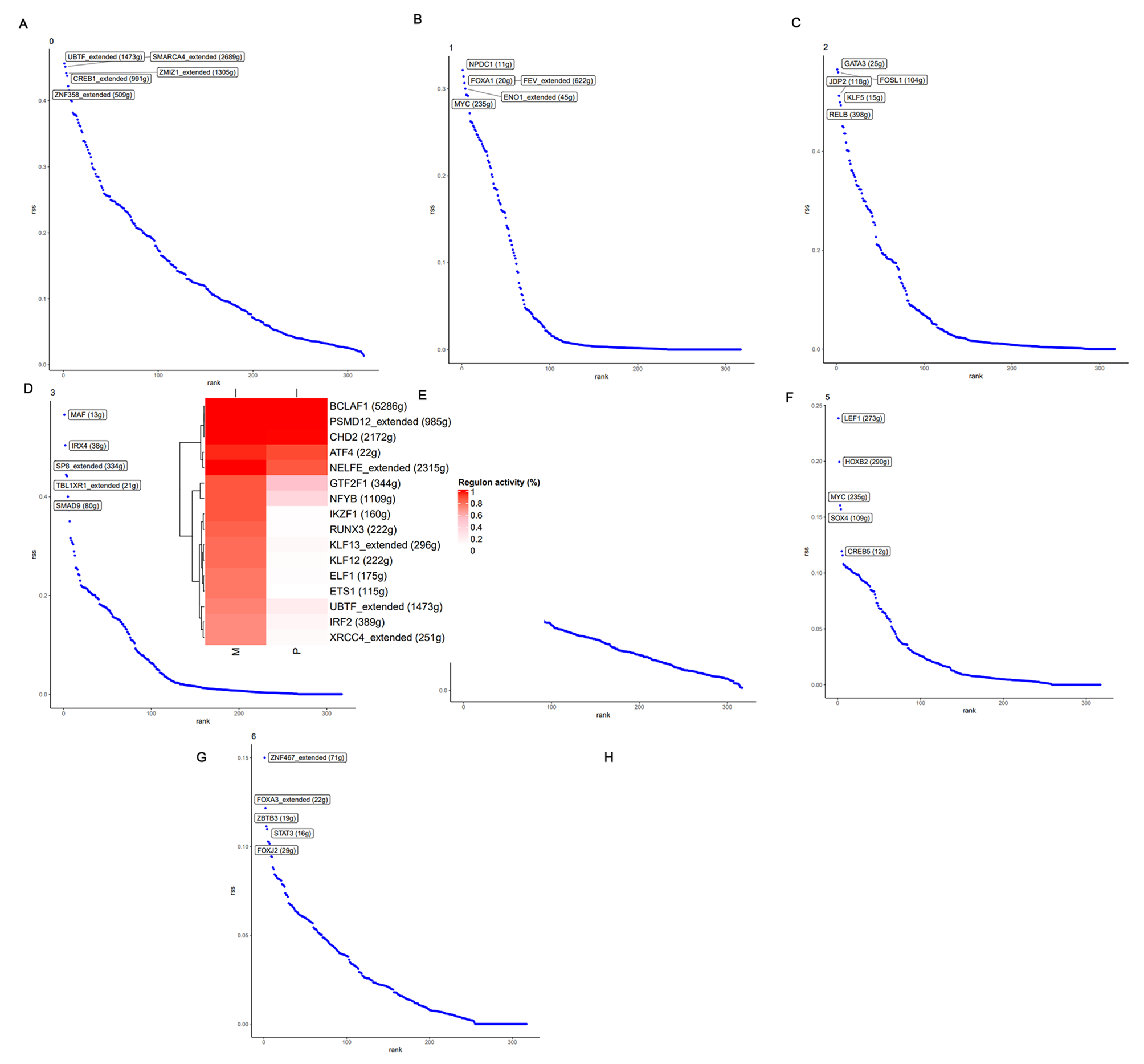

Supplement: Supplementary file 1 — Supplementary Fig. 1. (A–G). Transcription factors activated by various luminal cell subsets; (H). Transcription factor activity comparison between primary and lymphatic metastatic lesions. [file 40164_2023_407_MOESM1_ESM.png]

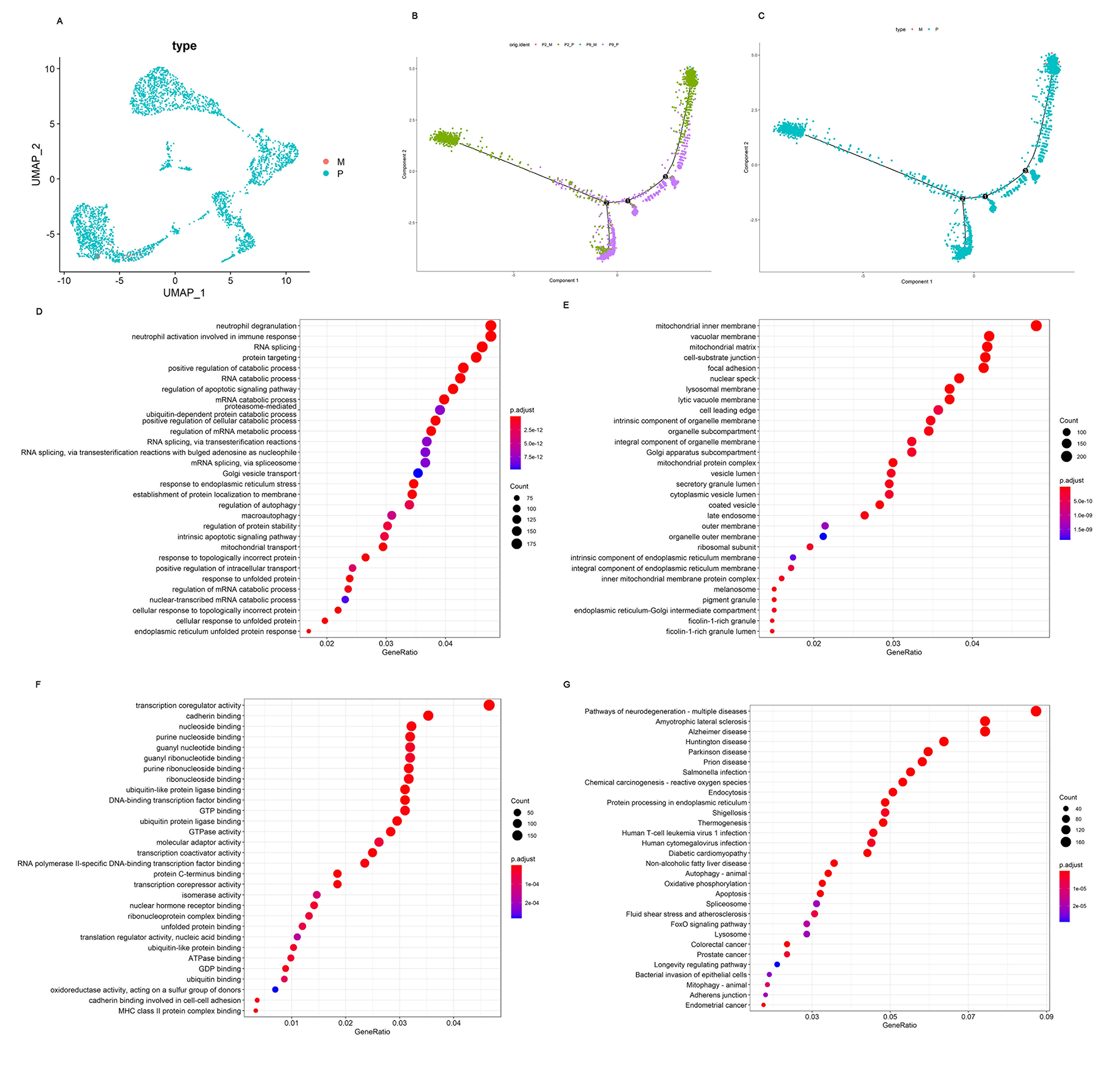

Supplement: Supplementary file 2 — Supplementary Fig. 2. (A) Luminal cell distribution in primary and lymphatic metastatic lesions demonstrated using uniform manifold approximation and projection (UMAP) analysis. (B–C). Monocle 2 trajectory plot showing dynamics of luminal subclusters of primary and lymphatic metastatic lesions; (D). Functional enrichment analysis of DEGs in luminal (GO-BP); (E). Functional enrichment analysis of DEGs in luminal (GO-CC); (F). Functional enrichment analysis of DEGs in luminal (GO-MF); (G). Functional enrichment analysis of DEGs in luminal (KEGG). [file 40164_2023_407_MOESM2_ESM.png]

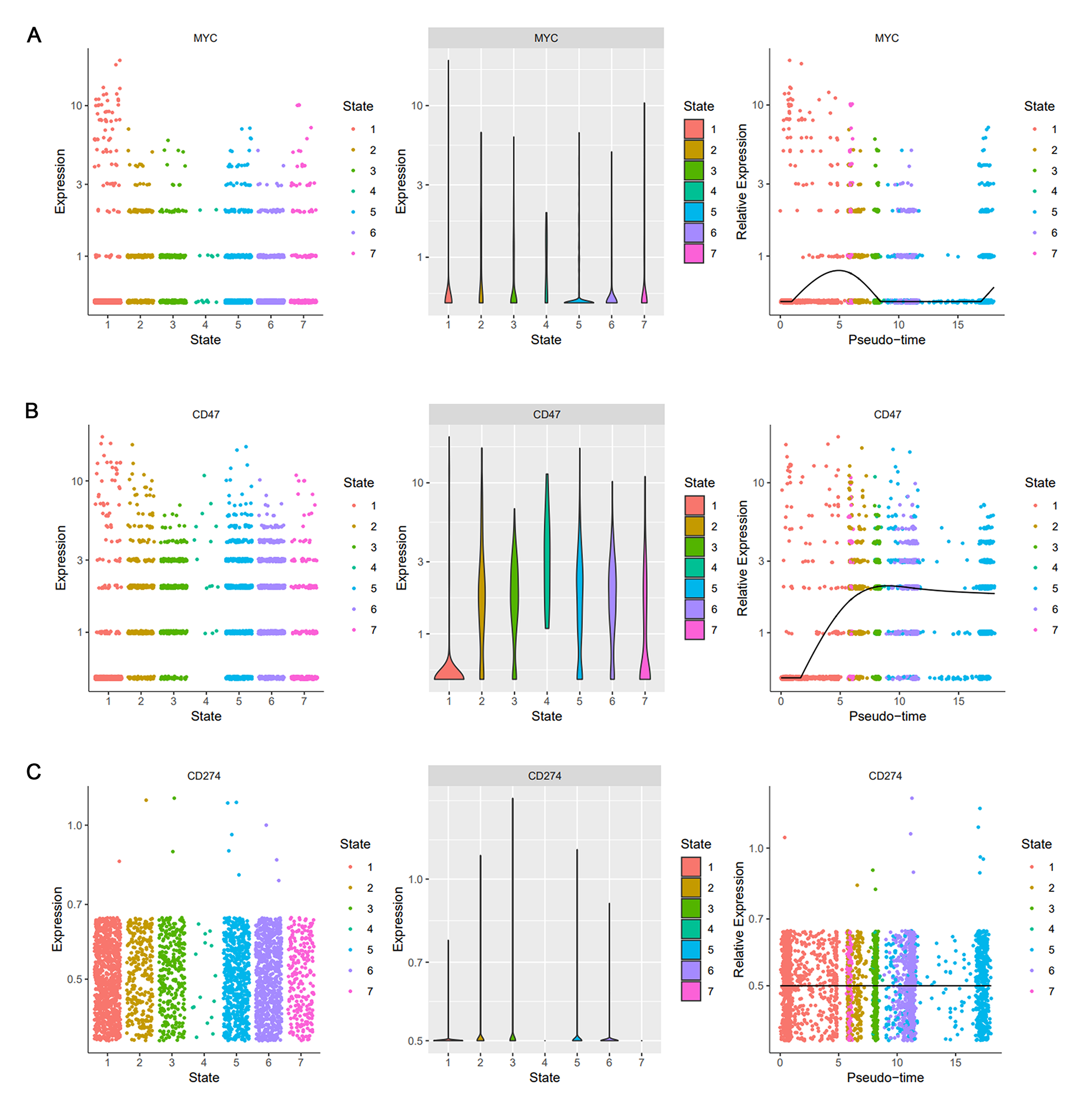

Supplement: Supplementary file 3 — Supplementary Fig. 3. Three representative genes with different expression patterns identified in the process of luminal cell differentiation: (A). MYC; (B). CD47; and (C). PDL1. [file 40164_2023_407_MOESM3_ESM.png]

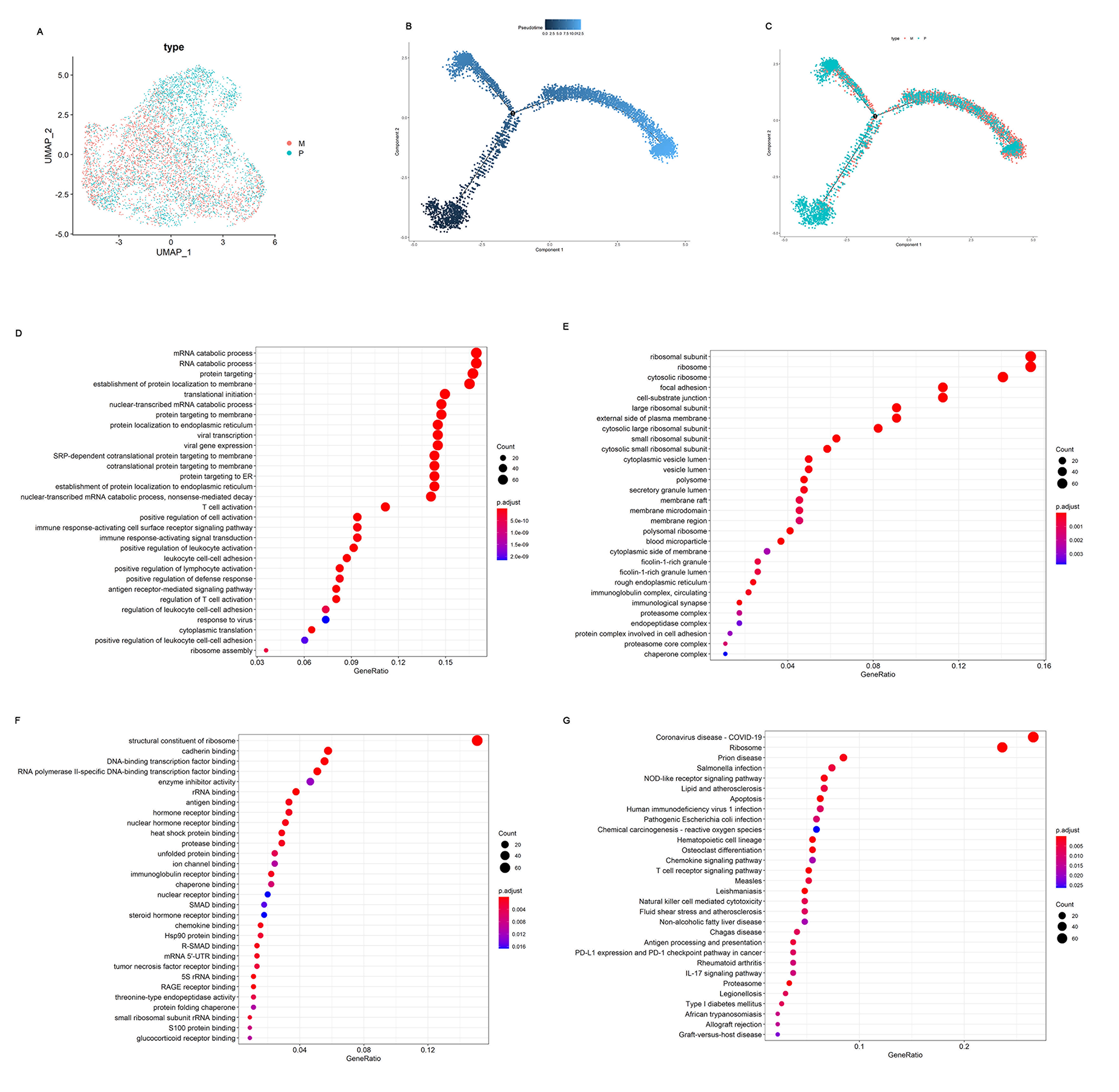

Supplement: Supplementary file 4 — Supplementary Fig. 4. (A). Distribution of CD + 8 T cells in primary and lymphatic metastatic lesions demonstrated using uniform manifold approximation and projection (UMAP) analysis. (B–C). Monocle 2 trajectory plot showing CD + 8 T cell dynamics in primary and lymphatic metastatic lesions; (D). Functional enrichment analysis of DEGs in CD + 8 T cells (GO-BP); (E). Functional enrichment analysis of DEGs in CD + 8 T cells (GO-CC); (F). Functional enrichment analysis of DEGs in CD + 8 T cells (GO-MF). (G). Functional enrichment analysis of DEGs in CD + 8 T cells (KEGG). [file 40164_2023_407_MOESM4_ESM.png]

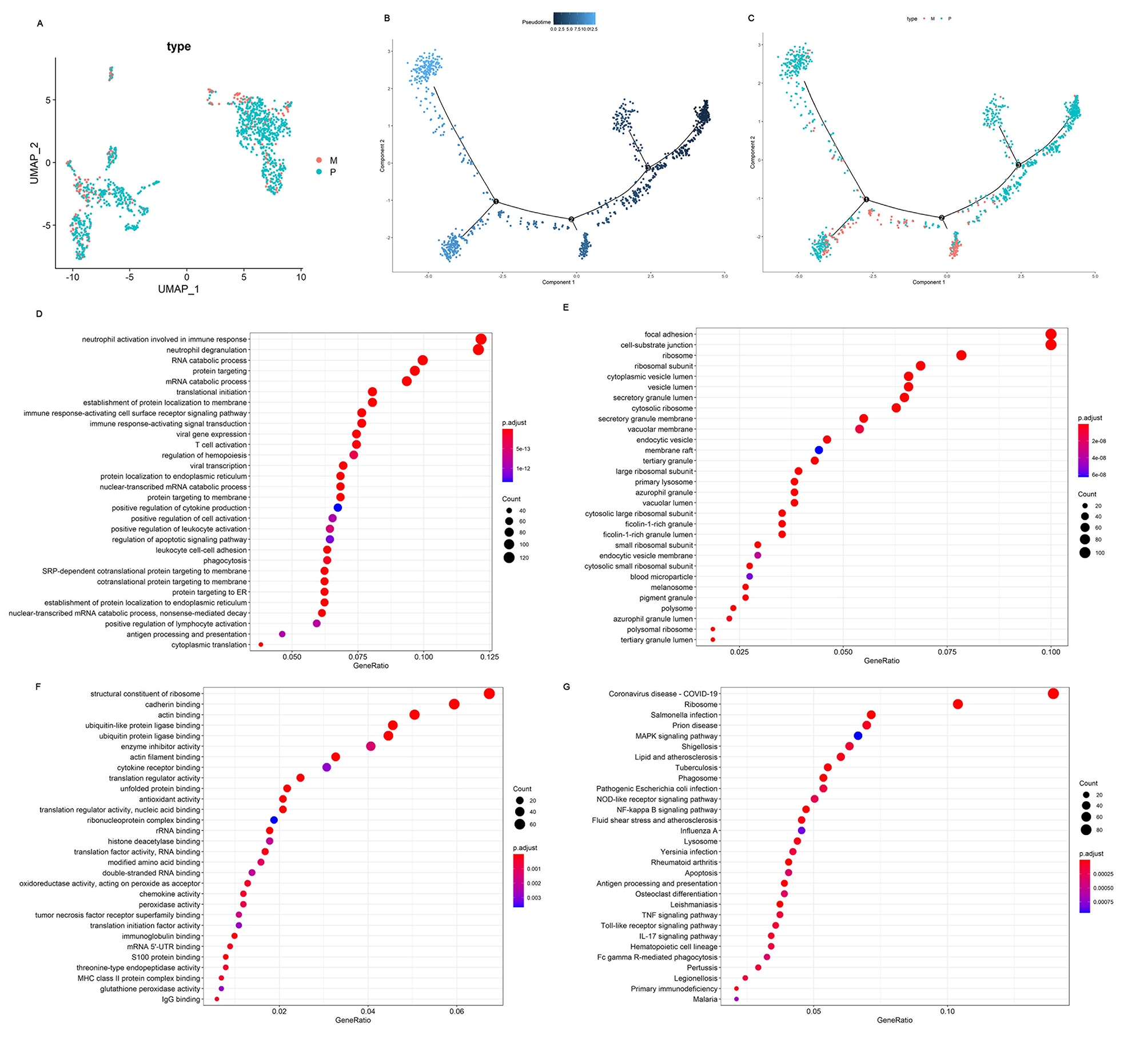

Supplement: Supplementary file 5 — Supplementary Fig. 5. (A). Monocyte distribution in primary and lymphatic metastatic lesions demonstrated using uniform manifold approximation and projection (UMAP) analysis; (B–C). Monocle 2 trajectory plot showing monocyte dynamics in primary and lymphatic metastatic lesions; (D). Functional enrichment analysis of DEGs in monocytes (GO-BP); (E). Functional enrichment analysis of DEGs in monocytes (GO-CC); (F). Functional enrichment analysis of DEGs in monocytes (GO-MF); (G). Functional enrichment analysis of DEGs in monocytes (KEGG). [file 40164_2023_407_MOESM5_ESM.png]

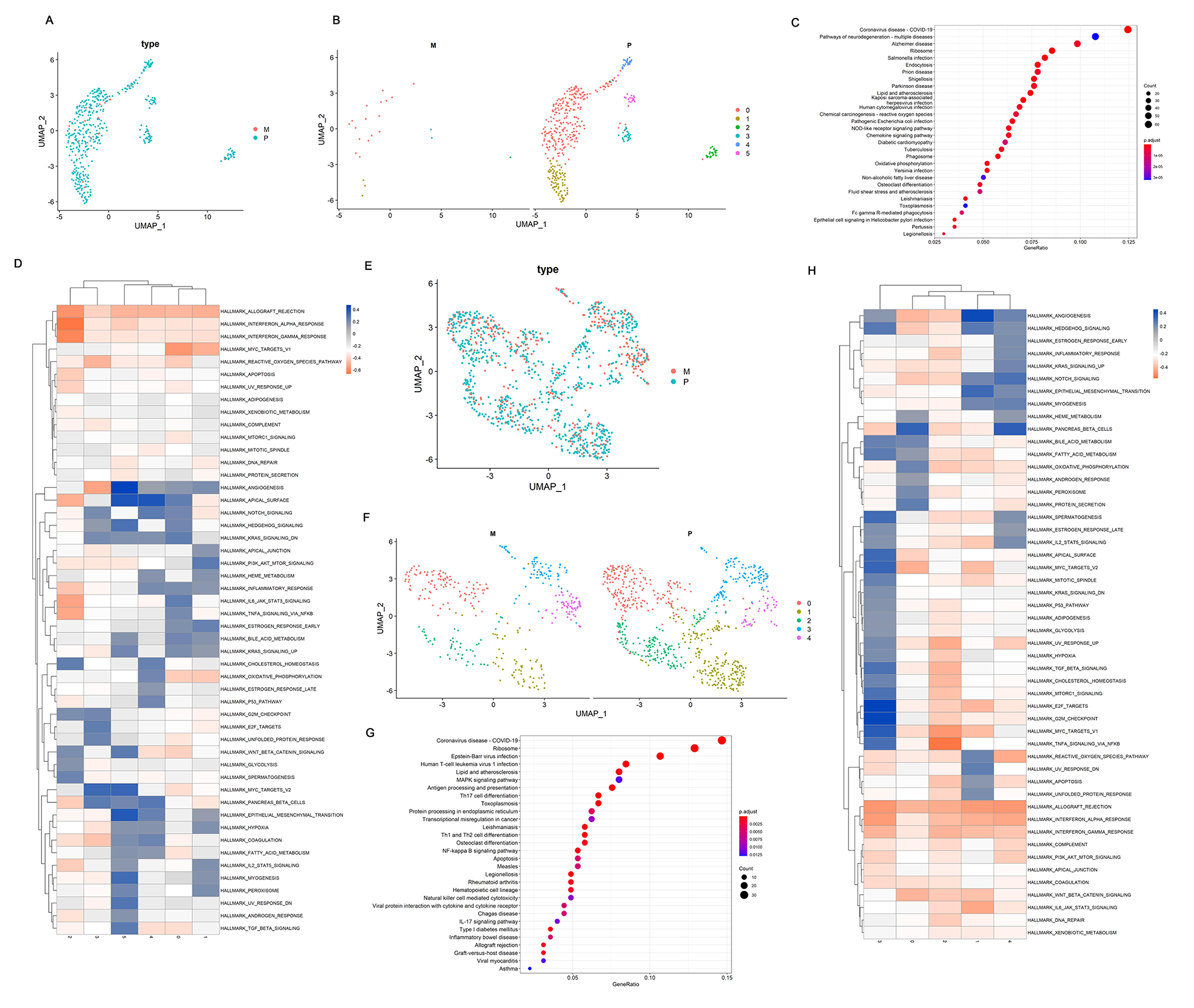

Supplement: Supplementary file 6 — Supplementary Fig. 6. (A–B). Distribution of neutrophil subclusters between primary and lymphatic metastatic lesions demonstrated using uniform manifold approximation and projection (UMAP) analysis; (C). Functional enrichment analysis of DEGs in neutrophils (KEGG); (D). GSVA heatmap of 50 hallmark gene sets in MSigDB database among neutrophil subclusters; (E–F). Distribution of NK subclusters between primary and lymphatic metastatic lesions demonstrated using uniform manifold approximation and projection (UMAP) analysis; (G). Functional enrichment analysis of DEGs in NK cells (KEGG); (H). GSVA heatmap of 50 hallmark gene sets in MSigDB database among NK cell subclusters. [file 40164_2023_407_MOESM6_ESM.png]

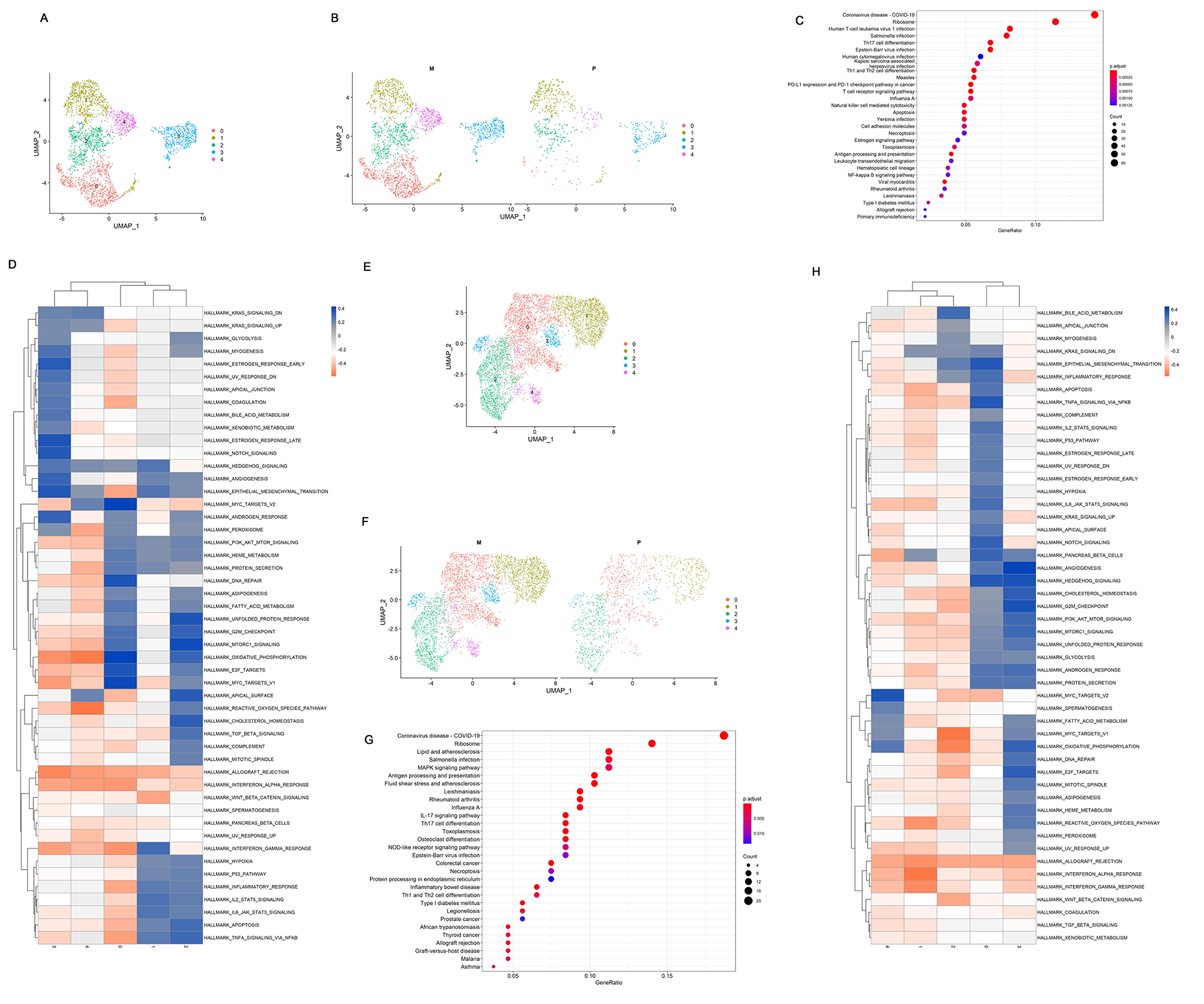

Supplement: Supplementary file 7 — Supplementary Fig. 7. (A–B). Distribution of Treg subclusters between primary and lymphatic metastatic lesions demonstrated using uniform manifold approximation and projection (UMAP) analysis; (C). Functional enrichment analysis of DEGs in Treg cells (KEGG); (D). GSVA heatmap of 50 hallmark gene sets in MSigDB database among Treg subclusters; (E–F). Distribution of Th subclusters between primary and lymphatic metastatic lesions demonstrated using uniform manifold approximation and projection (UMAP) analysis; (G). Functional enrichment analysis of DEGs in Th (KEGG); (H). GSVA heatmap of 50 hallmark gene sets in MSigDB database among Th subclusters. [file 40164_2023_407_MOESM7_ESM.png]

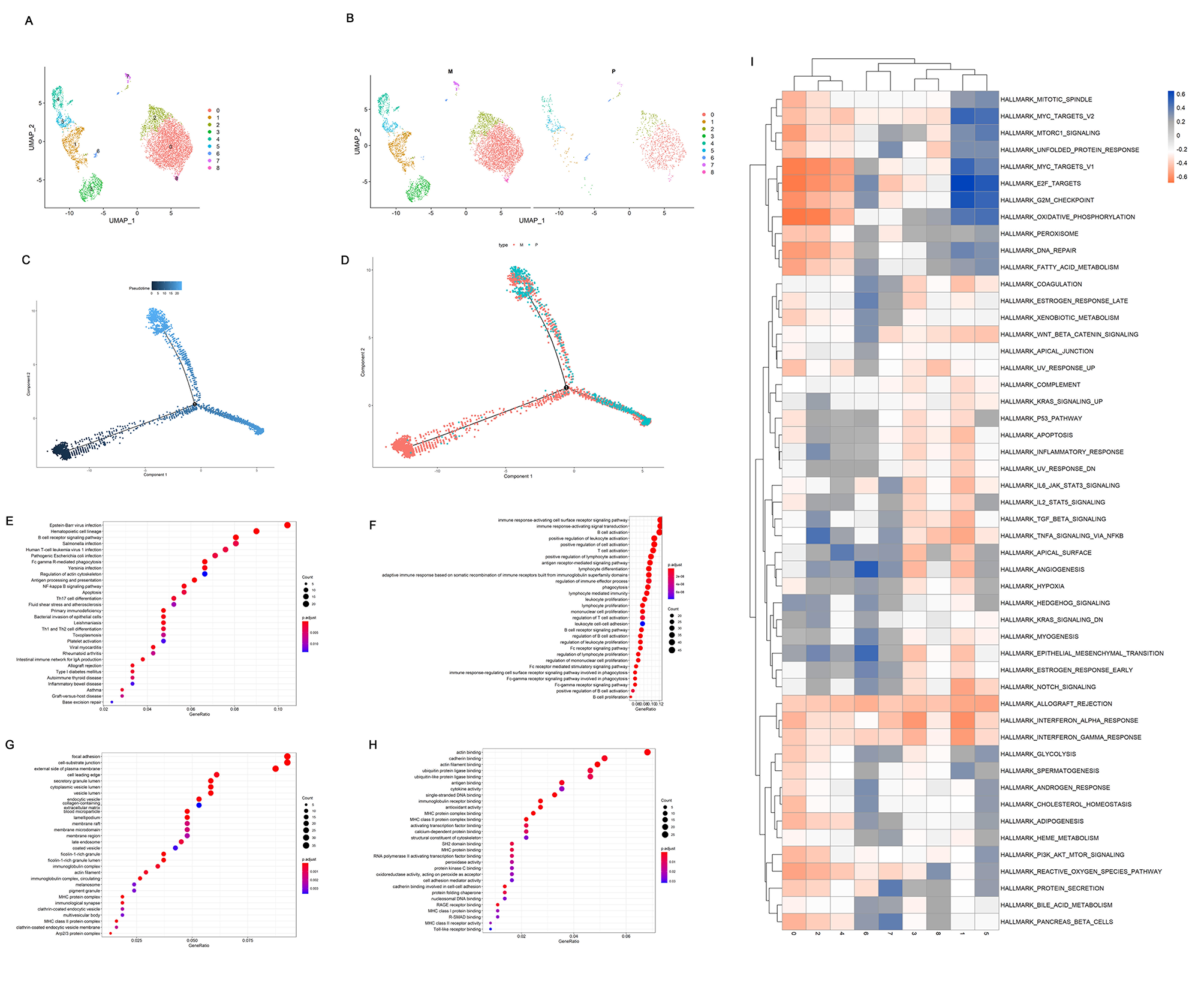

Supplement: Supplementary file 8 — Supplementary Fig. 8. (A–B). Distribution of B cell subclusters between primary and lymphatic metastatic lesions demonstrated using uniform manifold approximation and projection (UMAP) analysis; (C–D). Monocle 2 trajectory plot showing B cell dynamics in primary and lymphatic metastatic lesions; (E). Functional enrichment analysis of DEGs in B cells (GO-BP); (F). Functional enrichment analysis of DEGs in B cells (GO-CC); (G). Functional enrichment analysis of DEGs in B cells (GO-MF); (H). Functional enrichment analysis of DEGs in B cells (KEGG); (I). GSVA heatmap of 50 hallmark gene sets in MSigDB database among B cell subclusters. [file 40164_2023_407_MOESM8_ESM.png]

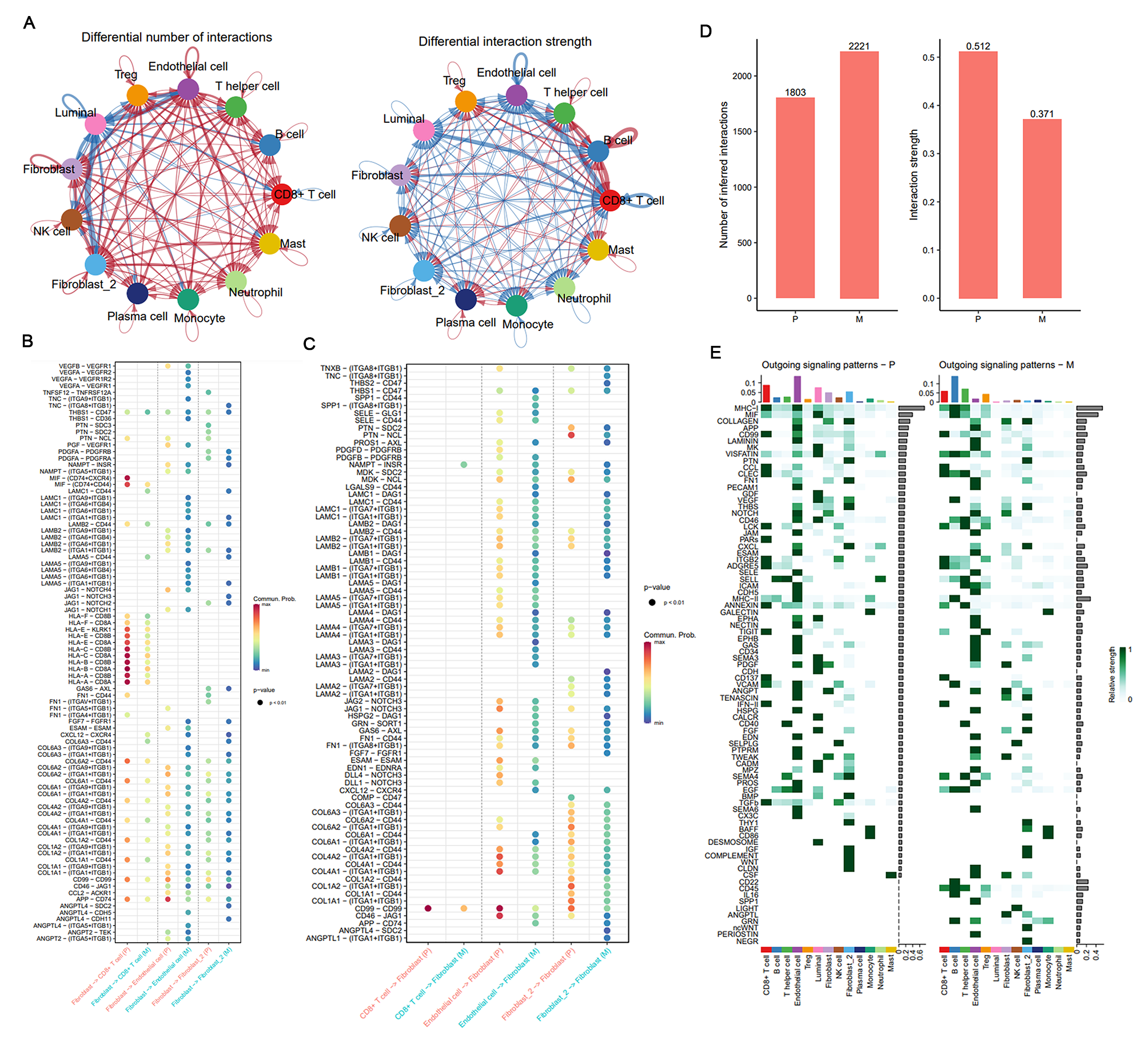

Supplement: Supplementary file 9 — Supplementary Fig. 9. (A). Cell communication network (including number and strength) in PCa; (B–C). Plot map showing increased signaling among cell types in primary and lymphatic metastatic lesions; (D). Bar chart showing the number of inferred interactions and interaction strength between primary and lymphatic metastatic lesions; (E). Outgoing signaling pattern of each cell type in primary and lymphatic metastatic lesions. [file 40164_2023_407_MOESM9_ESM.png]
